# Emphysematous conditions of the abdomen and pelvis: pearls and pitfalls

**DOI:** 10.1007/s00261-025-04997-7

**Published:** 2025-06-27

**Authors:** Ananya Panda, Mohamad Kayali, Yashant Aswani

**Affiliations:** University of Iowa Health Care, Iowa City, USA

**Keywords:** Emphysematous infections, Gas, Imaging, Computed tomography, Diabetes complications

## Abstract

Emphysematous conditions of the abdomen and pelvis are uncommon but are potentially lethal. A majority of these share common predisposing risk factors such as uncontrolled diabetes mellitus, immunocompromised states, chronic kidney disease, hepatic cirrhosis, malignancy, chemotherapy, and polytrauma. Computed tomography is the most utilized modality for the evaluation of emphysematous conditions in an emergency scenario. It is equally important to recognize mimics of the lethal emphysematous pathologies to avoid misdiagnosis. Gas-containing imaging mimics occur due to recent interventions, instrumentation, and fistulae. This review discusses the pertinent clinical and imaging features of emphysematous conditions of the abdomen and pelvis that can help confirm the diagnosis and guide patient management. For each of these emphysematous pathologies, we also discuss various gas-containing imaging mimics, as the management of these mimics differs from true emphysematous pathologies.

**Graphical abstract:**

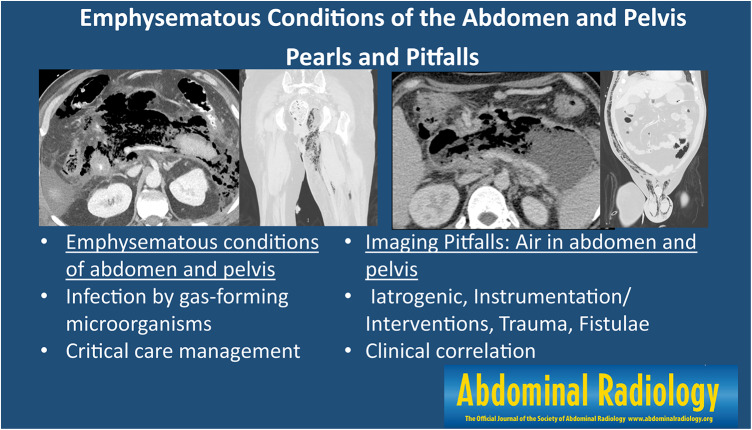

## Introduction

Emphysematous conditions of the abdomen and pelvis are uncommon but potentially life-threatening pathologies. These conditions are characterized by abnormal distribution of air due to infection by gas-forming microorganisms, or secondary to tissue necrosis or infarction [1]. These typically require urgent medical or surgical management, with a few exceptions. These conditions share predisposing systemic risk factors such as uncontrolled diabetes mellitus, immunocompromised states, chronic kidney disease, hepatic cirrhosis, malignancy, chemotherapy, and polytrauma [2]. Diabetes mellitus is the most common predisposing condition, as hyperglycemia is associated with lowered immune response, increased microbial colonization and fermentation (that releases carbon dioxide and nitrogen), and chronic vascular insufficiency or neuropathy (which predisposes to tissue ischemia and necrosis) [3].

Emphysematous infections are often polymicrobial and admixed with aerobic and anaerobic flora; common organisms include *Klebsiella pneumoniae*, *Pseudomonas aeruginosa*, *Clostridium* species, *Escherichia coli*, *Bacteroides* species, *Enterobacter* species, *Staphylococcus* species, *Streptococcus* species, and fungal infections such as *Candida* species, angioinvasive *Aspergillosis,* and *Mucormycosis* [1, 2]. Imaging mimics of emphysematous conditions occur due to recent interventions, instrumentation, and fistulae. However, contrary to the true emphysematous conditions, the management of these mimics greatly differs depending on the underlying etiology.

Computed tomography (CT) is the most commonly used imaging modality for diagnosis and can detect the presence of air more accurately compared to radiographs and ultrasound, particularly in the abdomen and pelvis, where gas is normally present inside the bowel. CT allows rapid delineation of the extent of involvement in an emergency setting and tailored protocols for follow-up. A few of these emphysematous conditions may, however, be first identified on abdominal radiographs and ultrasound. Air on ultrasound appears as echogenic foci with dirty posterior acoustic shadowing but may not be recognized due to a poor sonographic window, or may be mistaken for normal bowel gas [1]. In this review, we describe the spectrum of emphysematous conditions of the abdomen and pelvis, highlight various imaging mimics, and provide management recommendations. Table 1 summarizes these conditions and pitfalls.

**Table 1:** Emphysematous conditions of abdomen and pelvis and imaging pitfalls

Emphysematous conditions	Pitfalls
Emphysematous Gastritis	Benign Gastric Emphysema
Life-threatening Pneumatosis Intestinalis	Benign pneumatosis Pneumatosis cystoides
Emphysematous Pancreatitis	Pancreatitis with post-intervention changes Pancreatitis with enteric fistula
Emphysematous Cholecystitis	Air-containing gallstones Pneumobilia
Emphysematous Hepatitis/ Hepatic Gas gangrene	Hepatic abscess Post-locoregional therapy changes
Emphysematous Pyelonephritis	Emphysematous Pyelitis Post-intervention changes Bowel Fistula
Emphysematous Cystitis	Bladder Trauma Intramural Fat
Emphysematous Aortitis	Aorto-enteric fistula Post-Intervention/Surgery changes
Retroperitoneal Fasciitis/ Emphysematous Iliopsoas abscess	Pneumoretroperitoneum
Emphysematous Prostate Abscess	Infected SpaceOAR Urosymphyseal Fistula
Gas Gangrene of Uterus	Post-partum/Procedural changes
Vaginitis Emphysematosa	Air in Vaginal Canal Vaginal Tampon
Necrotizing Fasciitis of Abdominal Wall/ Fournier gangrene	Benign Subcutaneous Emphysema Trauma
Emphysematous Epididymo-Orchitis	Intratesticular echogenic foci Inguinoscrotal hernia with bowel Malignancy

## Emphysematous gastritis

Emphysematous gastritis (EG) occurs due to invasion of the gastric mucosa by gas-forming organisms and represents a true emphysematous infection of the gastric wall [1]. Apart from underlying systemic risk factors, gastric mucosal damage from prior surgery, malignancy, ingestion of corrosive agents, alcohol abuse, chemotherapy, and nonsteroidal ant-inflammatory drugs (NSAIDs) predispose to invasion by gas-forming organisms [1, 4, 5]. Recently, EG has also been reported with COVID-19 infection [6]. Patients typically present with abdominal pain and signs of sepsis, hematemesis, and vomiting [5].

**Fig. 1:**
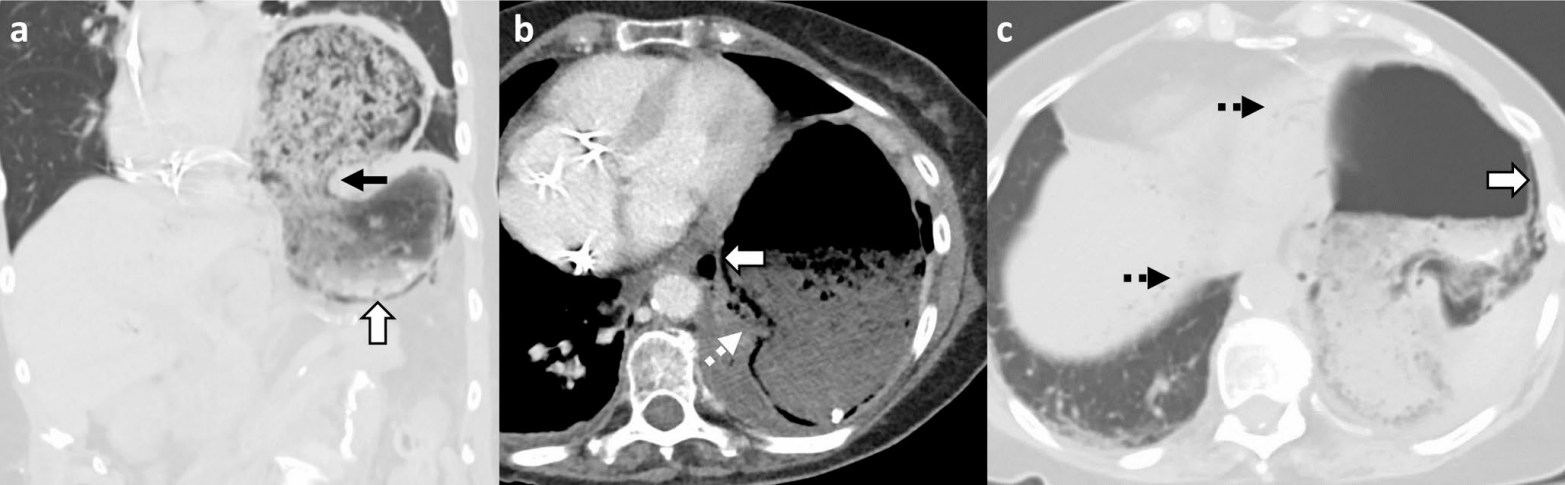
Emphysematous Gastritis. An 83-year-old diabetic patient with right upper quadrant pain and elevated serum lactate. CT images show gastric herniation with volvulus (black arrow, **a**), air in the gastric wall (white block arrow, **a**–**c**), gastric wall thickening (dotted white arrow, **b**), and portal venous gas (dotted black arrows, **c**). Conservative management was attempted due to advanced age. The patient died a day after the CT

*Imaging*: On radiographs, EG appears as linear or bubbly lucencies outlining the gastric wall. A definitive diagnosis is provided by CT. On CT, EG is characterized by linear streaky or bubbly air outlining the gastric wall with gastric wall thickening and perigastric inflammatory changes (Fig. 1). Air within the gastric wall is best seen on lung window or broad window CT settings and should be distinguished from mottled ingested food debris within the gastric lumen. There can be concomitant portal venous gas, pneumatosis intestinalis, and pneumoperitoneum [1, 2].

*Prognosis and management:* Historically, EG was associated with a high mortality rate of 60–80%, but mortality has decreased to < 50% in recent years [2, 7]. There is an increasing trend for conservative management as patients often have multiple comorbidities precluding surgery [7–9]. Management includes intravenous fluid resuscitation, broad-spectrum antibiotics covering Gram-negative and anaerobic organisms, empiric antifungal coverage, proton pump inhibitors, and nasogastric decompression with nil per oral (NPO) status [8, 9]. Esophagogastroduodenoscopy (EGD) shows friable gastric mucosa with air bubbling through the mucosa [10]. EGD with biopsy and culture confirms the diagnosis and guides appropriate antibiotic therapy. Surgery is reserved for patients with uncontrolled sepsis, strictures, gastric infarction, or gastric perforation [5]. However, the prognosis remains poor after surgery.

### Imaging pitfall of emphysematous gastritis

#### Benign gastric emphysema

**Fig. 2:**
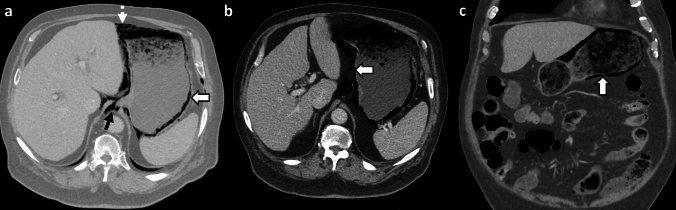
Benign gastric emphysema. A 78-year-old with severe retching, epigastric pain, and Mallory-Weiss tear of the esophagus. CT images show pneumomediastinum at the gastroesophageal junction (black arrow, **a**), extensive gastric wall emphysema (white block arrow,** a**–**c**), and pneumoperitoneum (dotted white arrow, **a**) tracking down from pneumomediastinum. There is a distinct lack of perigastric inflammation despite extensive gastric emphysema. Serum lactate and WBC counts were normal. The patient was managed conservatively with bowel rest and recovered fully

Benign gastric emphysema (BGE) refers to air within the gastric wall with normal gastric wall thickness (< 3 mm) and absence of perigastric inflammatory changes [2]. Similar to EG, BGE can be associated with portal venous gas, benign pneumatosis intestinalis, and pneumoperitoneum [7] (Fig. 2). However, in contrast to EG, patients with BGE are not toxic and have normal WBC counts and serum lactate levels. EGD with biopsy and culture excludes gastric necrosis and infection. BGE is caused by violent coughing, retching, vomiting, or ingestion of hydrogen peroxide [7]. BGE is also associated with chronic obstructive pulmonary disease and asthma, wherein air can dissect down from the mediastinum [7]. Management comprises decompression with a nasogastric tube and bowel rest with good patient outcomes. Morbidity (< 30%) is chiefly attributed to other co-morbid conditions and underlying systemic illness [7].

## Pneumatosis intestinalis

Presence of extraluminal, mural gas within the wall of the small bowel is called pneumatosis intestinalis (PI); when present in the colonic wall, it is referred to as pneumatosis coli. Life-threatening PI is associated with bowel obstruction (especially strangulation), mesenteric ischemia, toxic megacolon, intestinal infection, necrotizing enterocolitis, caustic ingestion, and molecular targeted therapy [11, 12]. Patients may present with abdominal pain, diarrhea, vomiting, and signs of peritonitis. Laboratory tests can show marked leucocytosis with neutrophilia, elevated hematocrit, metabolic acidosis (serum lactate > 2.0 mmol/L, serum bicarbonate < 20 mmol/L, blood pH < 7.3), and elevated serum amylase level > 200 U/L [13].

**Fig. 3:**
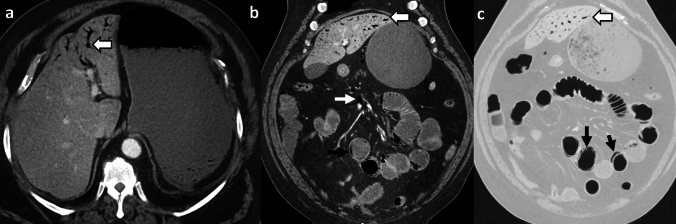
Life-threatening pneumatosis intestinalis. A 47-year-old with sudden-onset abdominal pain. CT images show portal venous gas (white block arrows, **a**–**c**) in peripheral liver, gas in the superior mesenteric vein (white arrow, **b**), and dilated small bowel with pneumatosis intestinalis (black arrows **b**, **c**). Serum lactate and WBC counts were elevated, and the patient was taken for laparotomy. Intraoperatively, there was dilation of the jejunum with evidence of ischemia

*Imaging:* On radiographs, air may be seen as linear or rounded lucencies along the bowel wall. On CT, life-threatening PI is associated with abnormal bowel wall thickness, abnormal bowel enhancement, bowel dilation, mesenteric fat stranding, mesenteric vascular attenuation or occlusion, ascites, and porto-mesenteric venous gas [14] (Fig. 3).

*Prognosis and management:* Patients with life-threatening PI on imaging, peritoneal signs, and lactic acidosis are managed with emergent exploratory laparotomy [15]. In the absence of these factors, medical management with bowel rest, antibiotics, and inhalational oxygen therapy may be considered [16]. Endovascular interventions with superior mesenteric artery revascularization are being increasingly performed for acute mesenteric ischemia in hemodynamically stable patients without overt peritonitis. Compared to open surgery, an endovascular approach is associated with a lower bowel resection rates and a lower 30-day mortality [13]. The risk of mortality is highest in patients older than 60 years of age and sepsis [16].

### Imaging pitfalls of pneumatosis intestinalis

#### Benign pneumatosis intestinalis and benign pneumatosis coli

**Fig. 4:**
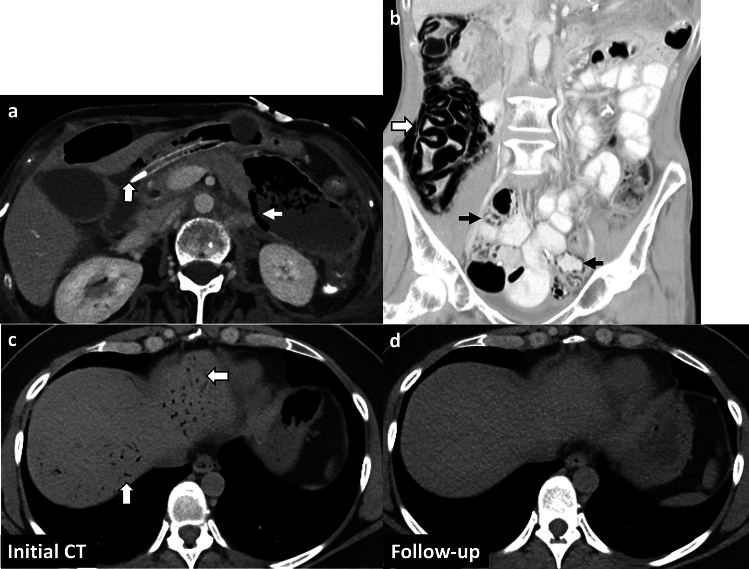
Benign pneumatosis intestinalis. **a** Pneumatosis intestinalis associated with feeding jejunostomy. A 54-year-old with breast cancer and disseminated metastases on feeding jejunostomy (white block arrow). The jejunal loop is dilated with pneumatosis (white arrow), which was unchanged on multiple CTs over 3 months. Serum lactate was normal at all time points, confirming benign pneumatosis intestinalis. **b** Pneumatosis intestinalis and coli associated with molecular targeted therapy. A 56-year-old with renal cancer on cabozantinib therapy. There is benign pneumatosis intestinalis (black arrows) and pneumatosis coli (white block arrow). Findings were unchanged over 6 months. **c**, **d** Portal venous gas associated with accidental hydrogen peroxide ingestion. A 39-year-old with epigastric pain, vomiting, and foaming at the mouth. Initial CT (**c**) shows portal venous gas (white block arrow, **c**) concerning for mesenteric ischemia. Serum WBC and serum lactate were normal. The patient was treated with hyperbaric oxygen therapy, and findings completely resolved on follow-up CT (**d**)

Typically, these are seen as longer segments of bowel involvement, more rounded air lucencies, and more often associated with pneumoperitoneum, with a remarkable absence of peritoneal signs [17] (Fig. 4). Isolated pneumatosis coli is also more likely to be benign. Etiologies for benign pneumatosis include post solid organ transplant (particularly bilateral lung transplant), bone marrow transplantation, drugs (chemotherapy, molecular targeted therapy, steroids), pulmonary causes (e.g., COPD, mechanical ventilation, asthma, cystic fibrosis), iatrogenic (e.g. endoscopic procedures, feeding jejunostomy), gastrointestinal motility disorders (e.g. scleroderma), amyloidosis, and collagen vascular disease (e.g., systemic lupus erythematosus) [18]. Patients are asymptomatic or may have mild abdominal pain. Management includes treatment of the underlying cause, antibiotics, and inhalational oxygen therapy. Second-line therapy comprises short-term hyperbaric oxygen therapy for persistent symptoms, with surgery being reserved for complications and clinical worsening [18].

#### Pneumatosis cystoides intestinalis

**Fig. 5:**
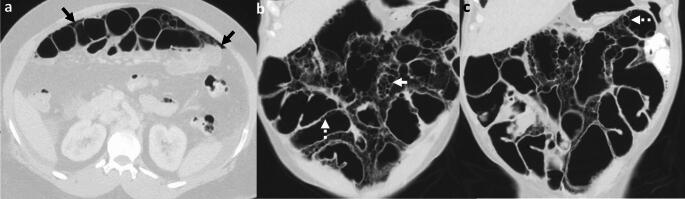
Pneumatosis cystoides intestinalis. A 57-year-old with systemic sclerosis, mild abdominal pain, and flu-like symptoms. CT images show pneumoperitoneum (black arrows, **a**) multiple submucosal cysts in the colon (solid white arrow, **b**) and small bowel (dotted white arrows, **b**, **c**). There was a distinct absence of peritoneal signs, and the patient was managed conservatively

This refers to benign submucosal or subserosal gas-filled cysts in the small bowel, colon (*pneumatosis cystoides coli)*, rectum, and mesentery [19] (Fig. 5). This condition is usually seen between 4 and 8th decades of life. Rupture of cysts leads to benign pneumoperitoneum. Associations include drug-related causes, ulcerative colitis, Crohn's disease, chronic intestinal pseudo-obstruction, sigmoid volvulus, COPD, AIDS, and systemic sclerosis [20]. When symptomatic, management includes hyperbaric oxygen therapy and treatment of the underlying cause [21].

## Emphysematous pancreatitis

Emphysematous pancreatitis (EP) is a severe form of acute necrotizing pancreatitis with gas replacing the necrotic pancreas and peripancreatic tissues. This develops due to infection by gas-forming enteric bacteria that translocate into the necrotic pancreatic tissue via the bloodstream, lymphatics, or through reflux from a patulous ampulla of Vater [1, 2].

**Fig. 6:**
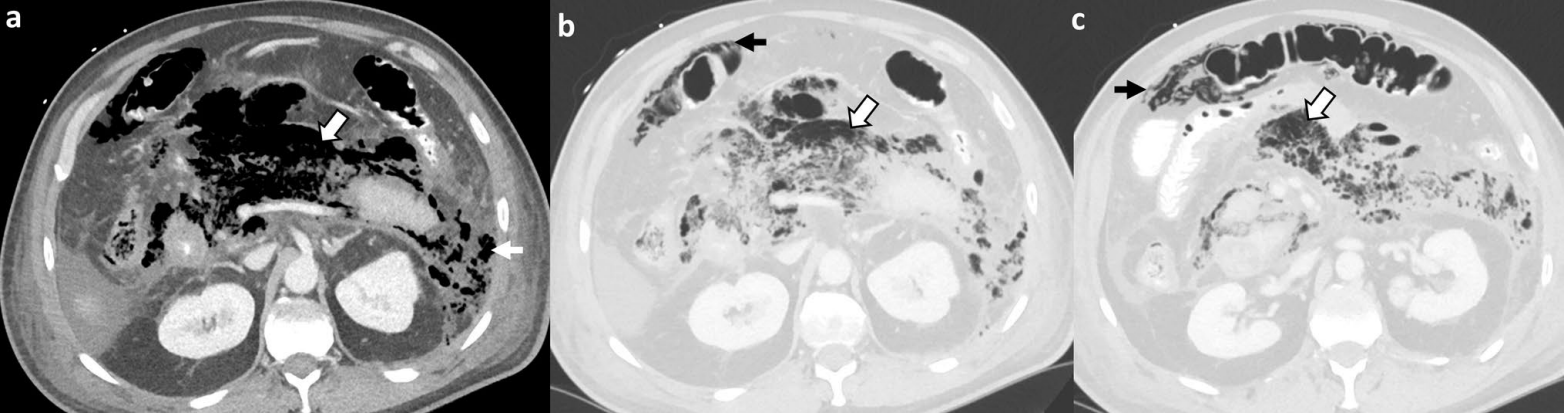
Emphysematous pancreatitis. A 64-year-old diabetic with alcohol abuse, with abdominal pain, elevated WBC counts, and elevated serum lactate levels. CT images show acute necrotizing pancreatitis with emphysema replacing the majority of the pancreas (white block arrows,** a**–**c**). Emphysema extends from the pancreatic bed/anterior pararenal space towards the left side (white arrow,** a**). There was also incidental benign pneumatosis coli in the transverse colon (black arrows, **b**, **c**), which resolved on follow-up CT

*Imaging:* Radiographs may show mottled lucencies in the mid-abdomen, but this is not specific. Ultrasound may have limited evaluation due to a poor acoustic window from overlying bowel and shadowing from gas within the pancreas [1]. CT is the modality of choice and can demonstrate the extent of necrotizing pancreatitis and identify vascular complications and organized collections amenable for drainage (Fig. 6). A portovenous phase CT suffices for diagnosis, however, multiphase CT or dual-energy CT protocol may be considered for assessment of pseudoaneurysm, hemorrhage, or thrombus [22]. In contrast to infected walled-off necrosis (WON), which contains scattered locules of air, EP shows a much greater extent of air replacing the pancreas and peripancreatic spaces.

*Prognosis and management:* Management comprises early endoscopic or percutaneous drainage, aggressive broad-spectrum antimicrobial therapy with an increasing role for delayed surgical intervention. The mortality ranges from 30%-50%, but has decreased with early interventions [23].

### Imaging pitfalls of emphysematous pancreatitis

#### Air within WON due to iatrogenic procedures or spontaneous fistula with stomach, colon, or small bowel

**Fig. 7:**
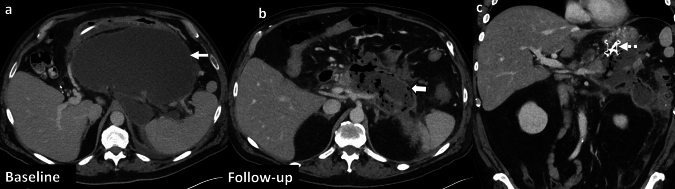
Air within necrotizing pancreatitis due to iatrogenic interventions. A 45-year-old with alcohol induced necrotizing pancreatitis. Baseline CT image (**a**) shows large walled-off necrosis (WON) replacing the pancreas (white arrow, **a**). This was treated with endoscopic cyst-gastrostomy using an Axios® stent. Follow-up CT images show decreased size of the WON with new post-procedural air (white block arrow, **b**) and the cyst-gastrotomy stent (dotted white arrow, **c**)

**Fig. 8:**
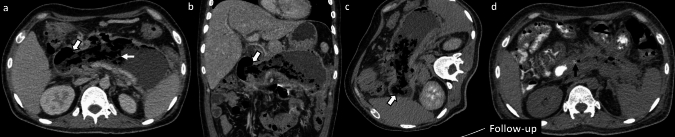
Air within necrotizing pancreatitis due to spontaneous enteric fistula. A 40-year-old with acute necrotizing pancreatitis. Initial CT images **a**–**c** show large WON replacing the entire pancreas with multiple air foci (solid white arrow, **a**). The WON is communicating with the duodenum (white block arrows, **a**, **b**). With right lateral decubitus (**c**), air and fluid gravitate into the second portion of the duodenum (white block arrow, **c**). Follow-up CT with enteric contrast **d** shows nearly resolved WON. The positive duodenal enteric contrast leaks into the pancreatic bed (black arrow, **d**)

Patients with acute necrotizing pancreatitis are often treated with percutaneous/endoscopic drainage procedures with or without necrosectomy as part of a minimally invasive “step-up” approach [24]. Acute pancreatitis can also be complicated by a spontaneous fistula with the hollow viscus due to bowel necrosis or ischemia. Amongst these, colonic fistula is the most common, followed by duodenal fistula [25]. The iatrogenic drainage or bowel fistula are characterized by presence of new or increasing air foci within the acute necrotizing collections/WON [26] mimicking EP (Fig. 7). CT with positive enteric contrast is useful for evaluation of pancreatic fistula with the gastrointestinal tract (Fig. 8). On CT, disruption of stomach or bowel wall, presence of extra-enteric contrast within the necrotic pancreas or within the percutaneous drains indicates an underlying enteric fistula [27]. To avoid misdiagnosis of EP or worsening infection, it is important to look for the history of recent interventions and correlate with serial WBC counts. Patients with endoscopic enteric drainage usually show improvement in both clinical and imaging findings. Comparison with serial CT scans shows progressive reduction of gas, a decrease in size of collections, and inflammatory changes. Conversely, increasing gas, increasing WBC trends, and clinical deterioration despite optimal percutaneous/ endoscopic drainage indicate developing infection [24].

## Emphysematous cholecystitis

Emphysematous cholecystitis (ECh) is a fulminant form of acute cholecystitis characterized by air within the wall of the gallbladder. This is more common in men between 5 and 7th decades of life. The etiopathogenesis is attributed to occlusion of cystic artery leading to ischemic changes and subsequent proliferation of gas-forming organisms in the wall of the gallbladder.

**Fig. 9:**
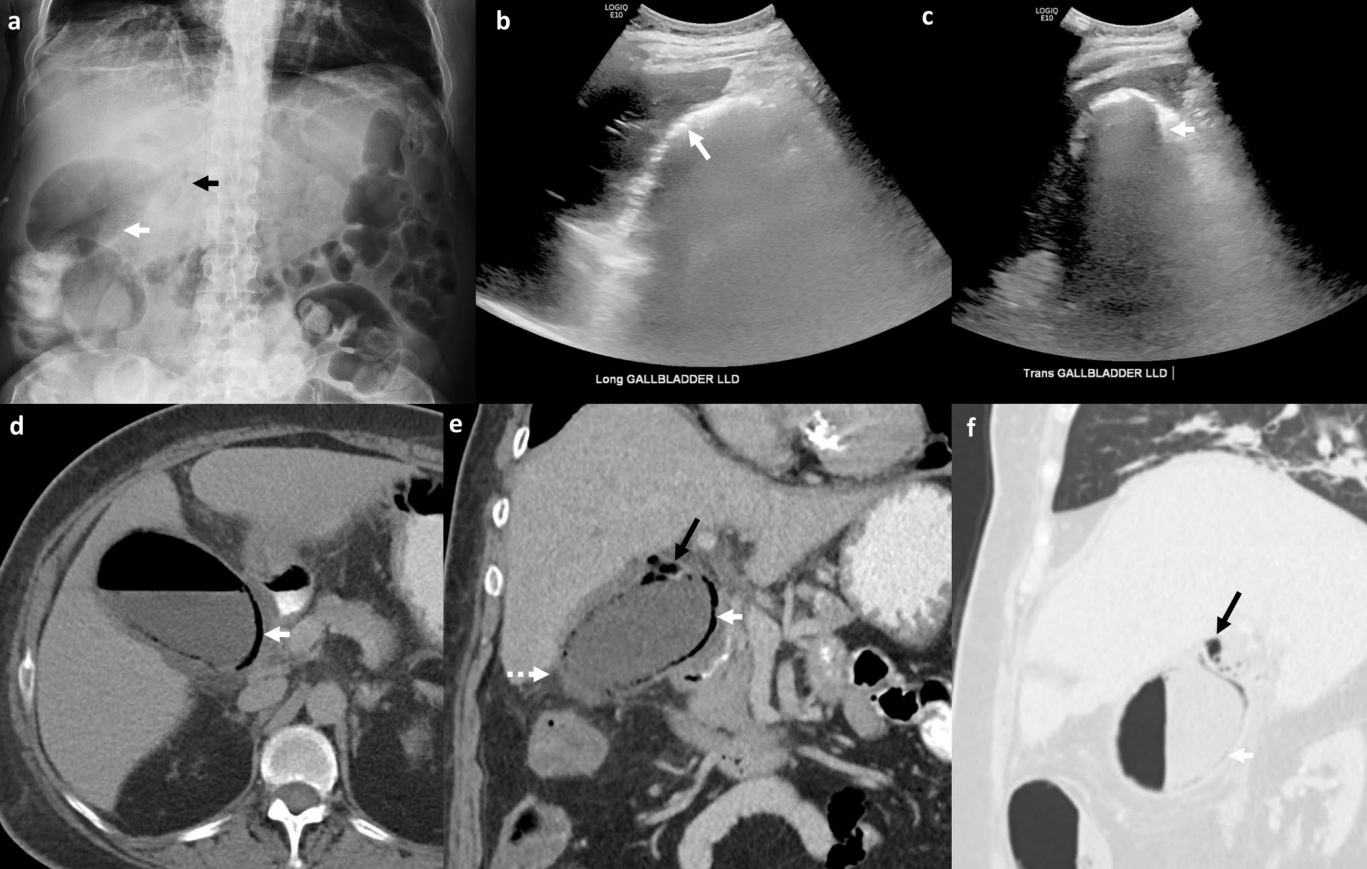
Emphysematous cholecystitis. A 54-year-old with diabetes mellitus and end-stage renal disease presented with right upper quadrant pain and sepsis. Abdominal radiograph **a** shows air in the right upper quadrant within the expected location of the gallbladder (white arrow, **a**) and abnormal curvilinear air in the right upper quadrant (black arrow,**a**). Longitudinal (**b**) and transverse (**b**) US images show curvilinear echogenic air foci in the gallbladder wall with dirty posterior acoustic shadowing (white arrows,** b**, **c**). CT images **d**–**f** show gallbladder wall thickening with air in the gallbladder wall (solid white arrows, **d**–**f**), air in the cystic duct (black arrows,**e**, **f**), and pericholecystic inflammation (dotted white arrow, **e**)

*Imaging:* On ultrasound, ECh is characterized by non-dependent air in the gallbladder with “dirty” posterior acoustic shadowing and real-time sonographic demonstration of non-dependent echogenic foci in the gallbladder lumen (“champagne” sign or “effervescent gallbladder” sign) [28, 29]. Sonographic Murphy’s sign may be false-negative due to underlying gangrene. On US, ECh may be mistaken for the wall-echo shadow complex of chronic cholecystitis [30]. CT is the next imaging investigation of choice and demonstrates the extent of involvement and complications. On CT, ECh is characterized by air in the gallbladder wall with or without air in the lumen (Fig. 9). Additional findings on CT include pericholecystic fat stranding, poorly enhancing gallbladder wall (which suggests gangrene), perforation, and cystic artery aneurysm or occlusion.

*Prognosis and management:* ECh can rapidly progress to gallbladder gangrene, perforation, and abscess. The mortality ranges from 15 to 25% [31]. Emergent cholecystectomy is the treatment of choice. If the patient is not a surgical candidate, emergent percutaneous cholecystostomy is a temporizing measure [32].

### Imaging pitfalls of emphysematous cholecystitis

#### Air-containing gallstones

**Fig. 10:**
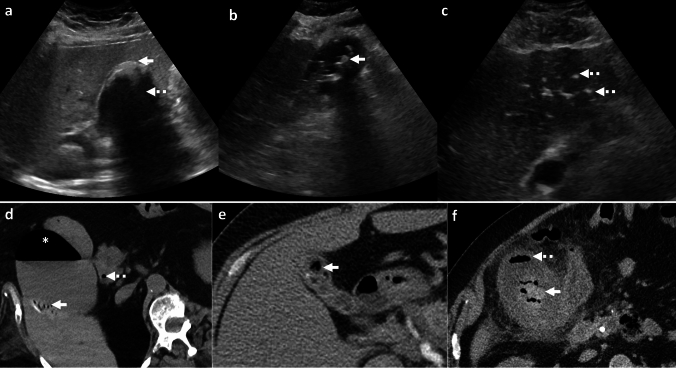
Pitfalls for emphysematous cholecystitis. **a** Wall echo shadow (WES) complex: Transverse US image shows chronic calculous cholecystitis and WES complex with thickened gallbladder wall (solid white arrow, **a**) and calculus with posterior acoustic shadowing (dotted white arrow, **a**). **b**, **c** Pneumobilia: Transverse US images **b**, **c** show echogenic air foci with dirty posterior acoustic shadowing within the lumen of the gallbladder (solid white arrow, **b**) and within the intrahepatic bile ducts (dotted white arrows, **c**). **d–f** Air-containing gallstones and pneumobilia in three different patients. CT image in (**d**) patient with trauma and abdominal pain shows distended gallbladder with gas in gallstones (solid white arrow, **d**), air-fluid level in gallbladder lumen (asterisk, **d**), and air in common bile duct post sphincterotomy (dotted white arrow, **d**). CT image of another patient **e** shows a collapsed gallbladder with incidental gas in gallstones (solid white arrow, **e**). CT after ERCP stenting in a patient with known acute cholecystitis **f** shows an air-fluid level in the gallbladder lumen (dotted white arrow, **f**) and air mixed with sludge and calculi in the gallbladder lumen (solid white arrow, **f**). There is no air in the gallbladder wall to suggest emphysematous cholecystitis

These are characterized by linear or star-shaped radiolucent shadows within the gallbladder lumen (“Mercedes-Benz” sign) (Fig. 10). The exact cause of air within these calculi is not known but is proposed to be due to trapped air within the fissures of the calculi or due to decaying bacteria within the calculi. These have no clinical significance other than a potential association with calculus cholecystitis [33].

#### Pneumobilia

Pneumobilia may be due to bilio-enteric fistula, post-ERCP, or percutaneous biliary interventions. Air is confined to the biliary tree and gallbladder lumen without any air in the gallbladder wall (Fig. 10).

## Emphysematous hepatitis

Emphysematous hepatitis (EH) or gas gangrene of the liver is a rare condition wherein the liver parenchyma is replaced by gas. This is attributed to a combination of hepatic necrosis due to compromise of both hepatic artery and portal venous supply with superadded infection by gas-forming organisms [2, 34]. Hepatic gas gangrene may be post-operative (e.g., liver transplant, biliary reconstruction surgeries, bilio-enteric anastomosis), post-traumatic after severe hepatic trauma, or spontaneous (due to malignancy and underlying immunosuppression) [35].

**Fig. 11:**
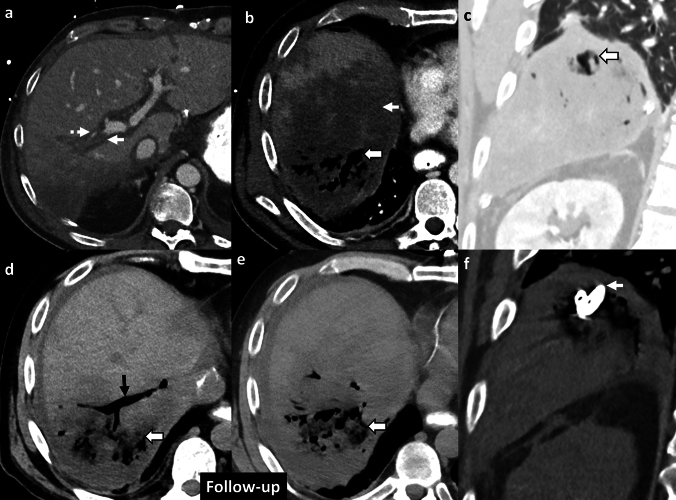
Emphysematous hepatitis or hepatic gas gangrene. A 43-year-old post-liver transplant patient with fever, sepsis, and abnormal liver function tests. Initial CT images **a**–**c** show thrombosed right portal vein (solid white arrow, **a**), diminutive right hepatic artery (dotted white arrow, **a**), hypoenhancing right hepatic lobe (solid white arrow, **b**) suggesting hepatic infarct with focal mottled air lucencies (block white arrow, **b**, **c**) within the infarcted liver consistent with hepatic gas gangrene. Note the distinct lack of fluid collection (block white arrow, **c**). Patient’s blood cultures were positive for *Enterobacter fecalis*, *Clostridium* species, and *Klebsiella* species. Follow-up CT images after one week **d**, **e** show increased extent of hepatic emphysema (block white arrows,** d**, **e**). Air also extends into the right hepatic vein (black arrow, **d**). Subsequently percutaneous drain was placed in focal emphysematous hepatitis (solid white arrow,** f**). The patient succumbed to sepsis despite aggressive measures, including redo surgery

*Imaging:* On CT, EH is characterized by a focal, sometimes lobar pattern of gas replacing liver parenchyma *without* a discrete fluid collection. There can be surrounding perfusion anomalies. CT is useful to demonstrate thrombosis of hepatic arteries and veins, and gas may also be seen extending to hepatic vessels (Fig. 11).

*Prognosis and management:* EH can rapidly progress to fulminant hepatic failure, if untreated. Management includes redo surgery, percutaneous drainage to control sepsis, and antibiotics [34]. However, mortality is nearly 100% despite optimal critical care and surgical management [35].

### Imaging pitfalls of emphysematous hepatitis

#### Liver abscess and infected biloma

**Fig. 12:**
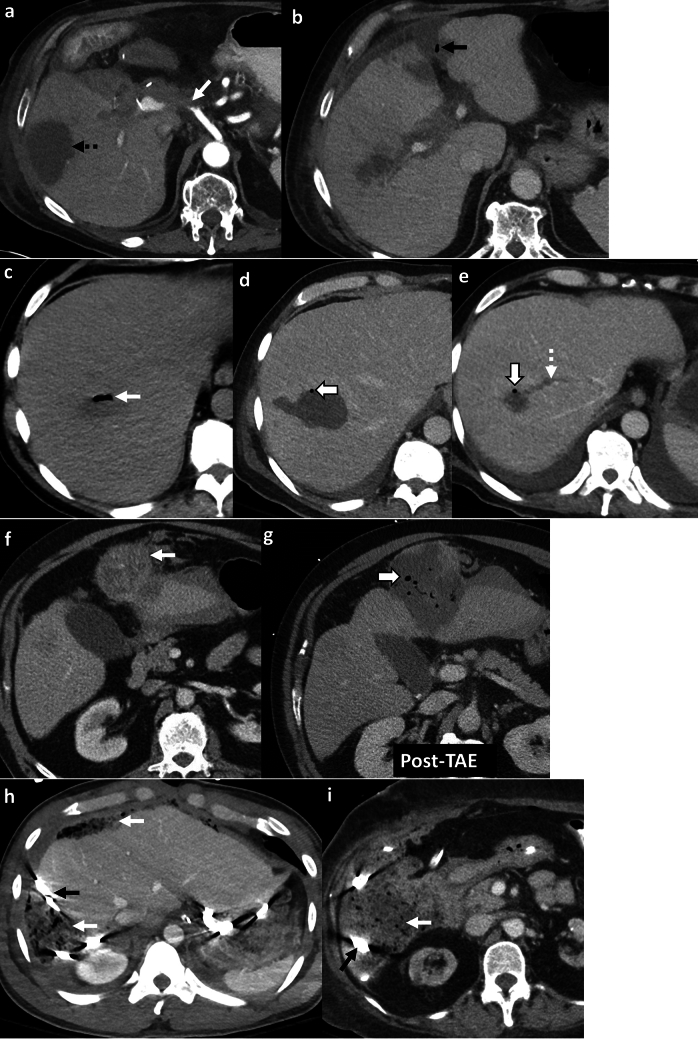
Pitfalls for emphysematous hepatitis. **a**, **b** Infected biloma: A 60-year-old post-Whipple’s procedure with fever and abnormal liver function tests. CT images show a thrombosed hepatic artery (solid white arrow, **a**), multiple bilomas (dotted black arrow, **a**), some with air (solid black arrow, **b**). **c**–**e** Post-ablation changes with infection: A 48-year-old post-Whipple’s procedure and hepatic metastasis ablation. Initial non-contrast CT (**c**) shows expected post-procedural air within the ablated region (solid white arrow, **a**). Follow-up CT at 3 weeks **d**, **e** shows increased size of ablated lesion with air locules (block white arrows, **e**, **f**) and a middle hepatic vein thrombus (dotted white arrow, **e**) extending to the inferior vena cava (not shown). A rapid increase in the size, increased fluid component, and sepsis were suggestive of hepatic abscess, which was confirmed on aspiration. **f**, **g** Post-embolization changes: A 63-year-old with hepatic cirrhosis and bleeding hepatocellular carcinoma (solid white arrow, **f**) treated with trans-arterial bland embolization (TAE). Post-TAE CT two days after embolization **g** shows expected post-procedural air foci in the treated lesion (block white arrow, **g**) with decreased enhancement due to tumor necrosis. Findings resolved on follow-up, and patient’s serial WBC counts were within normal limits. **h**, **i** Post-laparotomy sponges: A 60-year-old post-polytrauma damage control surgery with laparotomy sponges in perihepatic region and gallbladder fossa (solid white arrows **h**, **i**). Note radio-opaque ribbon markers (black arrows, **h**, **i**) indicating sponges

These are predominantly fluid-containing collections with air foci, in contrast to EH, which predominantly contains gas with minimal fluid (Fig. 12).

#### Post-ablation changes

In the early follow-up period, tiny air bubbles can be seen within the ablated cavities and along the percutaneous tracks. There may also be an increased size of the ablated lesion compared to pre-treatment imaging and adjacent perfusion changes. These are expected post-treatment changes due to tissue necrosis (Fig. 12). However, an increasing air on follow-up CT, fever, and increasing leukocytosis may indicate superimposed infection [36].

#### Post-embolization changes

In patients undergoing trans-arterial embolization for primary liver tumors or metastases, immediate post-embolization CT can show air foci within the treated lesion due to necrosis and intra-arterial injection of embolic agents [37] (Fig. 12). Some patients may present with post-embolization syndrome (PES) in the first week (usually within 2–3 days) after the procedure, characterized by fever, abdominal pain, nausea, vomiting and variable leukocytosis, mimicking infection. However, serial WBCs show an improving downward trend over time [38]. In contrast, superimposed infection with hepatic abscess usually presents 1–2 weeks after the embolization procedure, and serial WBCs show a worsening upward trend without treatment [39].

#### Laparotomy sponges and surgical hemostatic agents

These may be placed within the liver and in the perihepatic region after damage control surgery and other extensive surgeries. These mottled air lucencies can mimic emphysematous hepatitis. Laparotomy sponges can be recognized by the presence of a radiopaque ribbon marker (Fig. 12). For surgical hemostatic agents, it is important to correlate with the operative notes. These have a variable appearance on CT and are typically resorbed within 7–14 days [40].

## Emphysematous pyelonephritis

Emphysematous pyelonephritis (EPN) refers to a necrotizing gas-forming infection of the kidneys and perinephric tissues. This is more common in females with underlying systemic risk factors (e.g., diabetes mellitus, urinary tract calculus, chronic kidney disease, malignancy, and immunocompromised status). EPN occurs due to gas-forming urinary tract flora such as *Escherichia coli*, *Klebsiella pneumoniae*, *Pseudomonas aeruginosa*, and *Proteus mirabilis* [41]. Patients usually present with fever, flank pain, and dysuria.

**Fig. 13:**
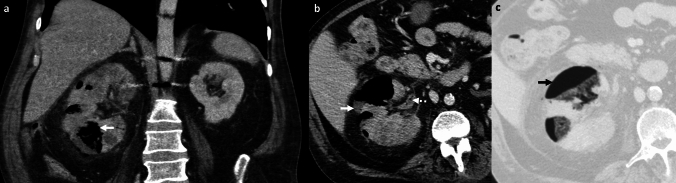
Emphysematous pyelonephritis. A 53-year-old with diabetes with fever, flank pain, and dysuria. CT images show an enlarged right kidney with striated nephrogram and air foci (white arrow, **a**). There is an extrarenal extension with an air-fluid level (white arrow, **b**). Air also extends to the renal pelvis with urothelial enhancement (dotted white arrow, **b**). Lung window images show emphysematous changes and air-fluid levels (black arrow, **c**). Findings are consistent with Class 3A emphysematous pyelonephritis (with perinephric extension). The patient was treated with antibiotics and percutaneous drainage

*Imaging:* Radiographs may demonstrate mottled lucencies within the renal parenchyma or crescentic air within the Gerota’s fascia. There may be additional retroperitoneal air outlining kidneys (“veiled kidney” sign) or linear streaks of air in paraspinal muscles. On US, the kidney may be enlarged with altered echogenicity with echogenic air foci within renal parenchyma [1]. CT establishes the diagnosis and demonstrates mottled air lucencies within the kidney, with or without renal abscess and extrarenal extension [2] (Fig. 13). Huang-Tseng classification is a clinico-radiological classification used to guide management and divides EPN into the following classes: Class 1: Gas in renal collecting system only; Class 2: Gas in the renal parenchyma without extension to extrarenal space; Class 3A: Extension of gas or abscess to perinephric space; Class 3B: Extension of gas or abscess to pararenal space; and Class 4: Bilateral EPN or EPN in solitary kidney [41].

*Prognosis and management:* Morbidity increases with Class 3 and Class 4 EPN and with one or more risk factors such as thrombocytopenia, acute renal function impairment, sepsis, or shock [41]. Most patients are treated conservatively with percutaneous drainage and antibiotics and are followed up with serial imaging. Nephrectomy is considered in patients who fail conservative management or in patients with extensive EPN and a fulminant clinical course. Mortality has decreased over the years and currently ranges between 10 and 40% [41, 42].

### Imaging pitfalls of emphysematous pyelonephritis

#### Emphysematous pyelitis

**Fig. 14:**
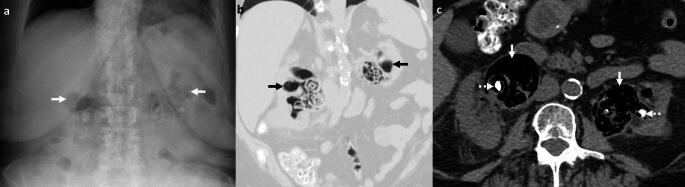
Emphysematous Pyelitis. A 42-year-old with renal calculi, dysuria, and no prior intervention. Abdominal radiograph (**a**) shows air in bilateral pelvicalyceal systems outlining renal calculi (white arrows, **a**). CT images show air localized to bilateral renal collecting systems and pelvis (black solid arrows, **b**, and white solid arrows, **c**) and outlining renal calculi (dotted white arrow, **c**). Note the absence of air in the renal parenchyma, excluding emphysematous pyelonephritis

This refers to air located within the renal collecting system only (Fig. 14). This is a relatively benign and self-limiting condition compared to EPN. Of note, Class 1 in the Huang-Tseng classification system refers to emphysematous pyelitis and can be managed with antibiotics [2].

#### Bowel fistula

**Fig. 15:**
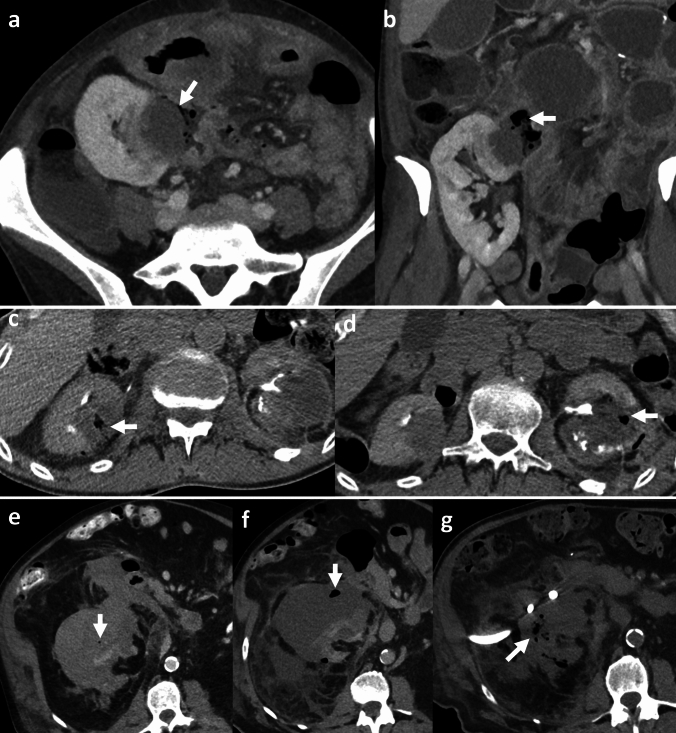
Pitfalls for emphysematous pyelonephritis. **a**, **b** Nephro-enteric fistula: A 35-year-old post renal transplant complicated by renal abscess and fistula with adjacent small bowel (white arrows, **a**, **b**). Air in the renal abscess is due to a bowel fistula. **c**, **d** Post-ablation changes. A 54-year-old with bilateral renal tumors treated with ablation on two different sessions. Post-procedure CT images show expected air foci in bilateral ablated regions (white arrows **c**, **d**), which resolved on follow-up. **e**–**h** Post-embolization changes with infection. A 71-year-old with end-stage renal disease and a spontaneous atraumatic right perirenal hematoma with active bleeding treated with right renal artery embolization. CT performed 3 days later shows (**e**) expected post-procedural small speck of air (white arrow, **e**). Follow-up CT at 6 weeks **f** shows worsening air foci and increased perinephric fat stranding suggestive of new emphysematous infection (white arrow, **f**). Follow-up CT at 3 months g after drain placement shows worsening air foci in the atrophied kidney (white arrow, **g**) and persistent perinephric inflammation. Right nephrectomy was thus performed. Drain contents showed *Pseudomonas aeruginosa*

Pyelonephritis may be complicated by bowel fistula (reno-duodenal, reno-enteric, or reno-colic fistula). Fistulization with the gastrointestinal tract may also be secondary to trauma and iatrogenic injuries after percutaneous nephrolithotomy [43, 44]. On CT, renal or perirenal collections with air foci, loss of fat planes with the adjacent hollow viscus, and disruption of adjacent bowel wall indicate a potential fistula (Fig. 15). CT with positive oral or rectal contrast is useful for demonstration of communication with the hollow viscus [27].

#### Post-renal interventions

Many renal interventions, such as renal mass ablation, renal artery embolization, and lithotripsy, are associated with postprocedural air within the kidney and perinephric region due to tissue necrosis and breakdown. Correlation with procedural notes and laboratory parameters is recommended to avoid misdiagnosis. In uncomplicated cases, postprocedural air resolves on follow-up. Increasing air or new air-fluid levels with clinical deterioration indicates a superimposed necrotizing infection (Fig. 15), necessitating appropriate treatment with antibiotics, percutaneous drainage, or nephrectomy in fulminant cases [45].

## Emphysematous cystitis

Emphysematous cystitis (ECy) refers to acute cystitis with air in the urinary bladder wall with or without air in the bladder lumen, and an associated perivesical inflammation. Occasionally, it may present in a more chronic form [1]. Predisposing factors include diabetes mellitus, urinary catheterization, neurogenic bladder, urinary retention, recurrent urinary tract infections, immunosuppression, and chemotherapy [46]. Most common causative organisms are *Escherichia coli* and *Klebsiella pneumoniae*; other organisms include *Proteus mirabilis*, *Enterobacter* species, *Streptococcus* species, and rarely fungal infection (*Candida* species and *Aspergillus*) [46]. The clinical features are variable, ranging from asymptomatic patients to sepsis [46].

**Fig. 16:**
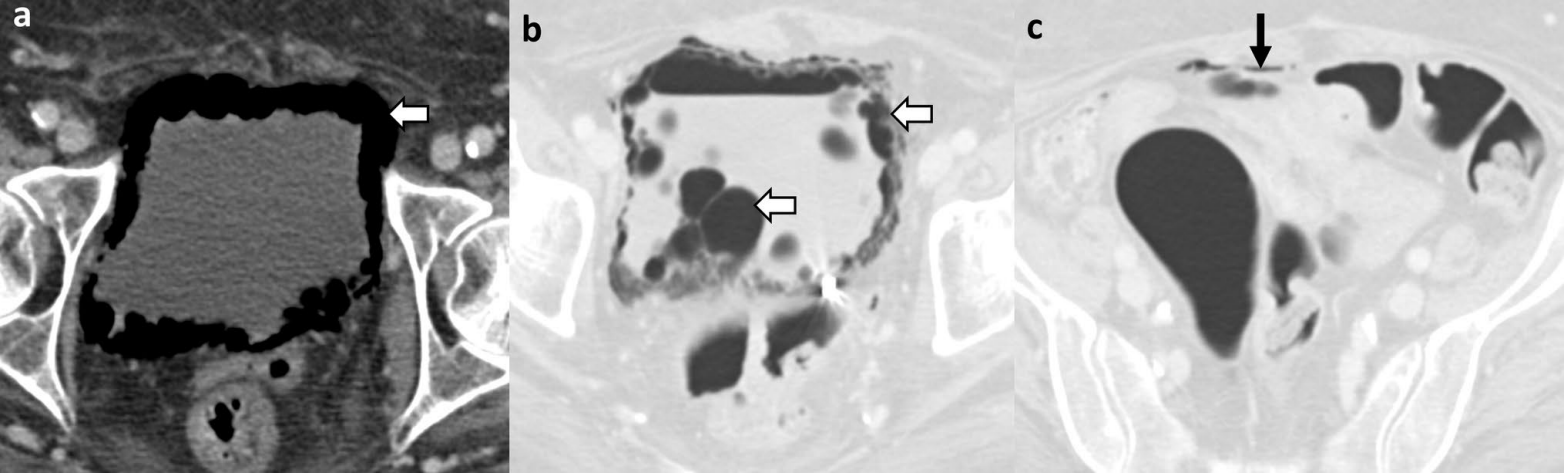
Emphysematous Cystitis. A 55-year-old diabetic patient with sepsis. CT images show extensive emphysematous cystitis (white block arrow, **a**, **b**) and trace pneumoperitoneum anteriorly (black arrow, **c**). Urinalysis was positive for urinary infection, and blood culture showed *Klebsiella pneumoniae*. The patient was managed with a Foley’s catheter and intravenous antibiotics but succumbed to sepsis

*Imaging:* On radiographs, ECy appears as mottled lucencies outlining the bladder wall [47]. CT offers accurate localization of air within the bladder wall and assessment of complications [48] (Fig. 16). Sonographic findings are non-specific due to limitations in detecting gas, however, findings include bladder wall thickening with echogenic foci due to air [1].

*Prognosis and management:* Treatment of ECy is generally conservative and requires antibiotics, bladder drainage and irrigation, and proper glycemic control. Surgery (debridement or cystectomy) is reserved for cases that fail conservative management or develop bladder perforation. Overall mortality is low (7–10%) compared to EPN[46].

### Imaging pitfalls of emphysematous cystitis

#### Urinary bladder injury

**Fig. 17:**
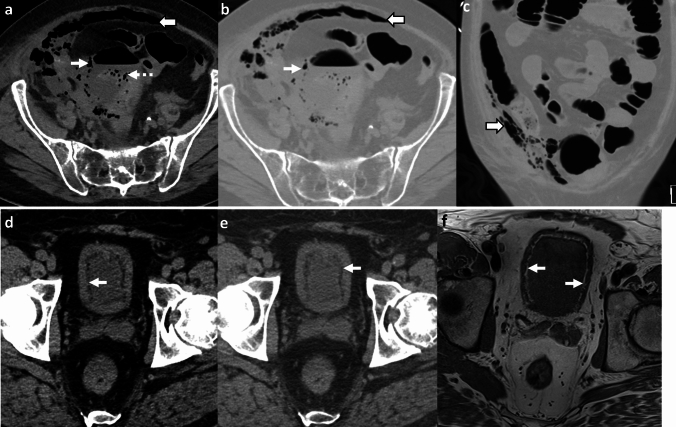
Pitfalls for emphysematous cystitis. **a**–**c** Urinary bladder injury. A 73-year-old with a newly diagnosed bladder tumor, post-transurethral resection of bladder tumor with pain and hematuria after the procedure. CT images show air in the right lateral bladder wall (solid white arrow, **a**, **b**), air within the tumor along the left lateral wall (dotted white arrow, **a**), and extensive extraperitoneal air (block white arrows, **a**–**c**). Findings are consistent with extraperitoneal bladder rupture. **d**–**f** Submucosal fat. A 54-year-old male patient with colon cancer underwent CT for staging. CT images show a low-density stripe along the anterior and lateral bladder walls (solid white arrows, **d**, **e**). T1-weighted (TIW) non-fat-saturated axial MRI image shows a hyperintense submucosal stripe (solid white arrows, **f**) which suppressed on fat-saturated imaging (not shown), confirming the presence of submucosal fat

Air in the bladder wall may also be seen in cases of traumatic or non-traumatic bladder injury (Fig. 17). Other mimics include air within the lumen or bladder wall due to fistula with bowel or vagina. These findings may be further assessed with CT cystography or CT with positive enteric contrast [48].

#### Submucosal fat

Fat in the urinary bladder wall is an incidental finding, more commonly seen in males due to chronic bladder inflammation. This appears as a low-density stripe along the anterior and lateral walls of the urinary bladder (Fig. 17). An excessive amount of fat in the bladder wall may be mistaken for ECy on CT but can be confirmed with proper windowing [49].

## Emphysematous aortitis

Emphysematous aortitis (EA) is a rare but life-threatening form of aortitis with fewer than 20 published case reports. Risk factors for EA include severe atherosclerosis, mycotic aneurysm, stent-graft repair, inflammatory bowel disease, colonic diverticulitis, and colon cancer [2, 50]. Most common organisms include *Salmonella* species and *Clostridium septicum* with underlying GI malignancy (e.g. colon cancer); other organisms include *Streptococcus* species, *Staphylococcus aureus*, *Escherichia coli*, and *Mycobacterium tuberculosis* [50–52]. Clinical findings are non-specific but include fever, chills, and sepsis.

**Fig. 18:**
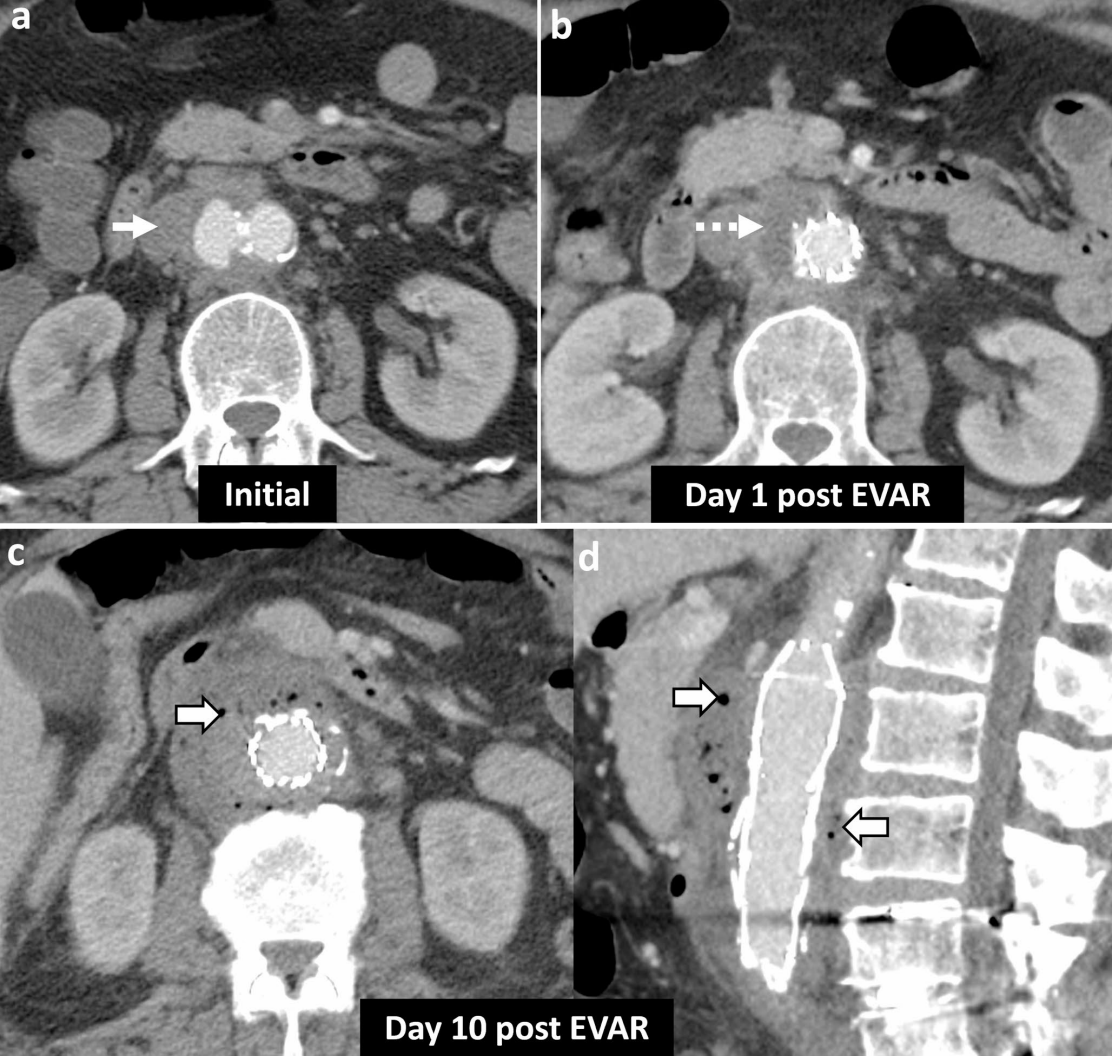
Emphysematous aortitis. A 73-year-old with pancreatic cancer and abdominal pain. CT at initial presentation **a** shows mycotic infrarenal aortic aneurysm with periaortic soft tissue and inflammation (solid white arrow, **a**). Day 1 post-EVAR CT **b** shows the expected aortic stent graft with peri-aortic soft tissue (dotted white arrow,** b**). Note the distinct absence of air on post-operative day 1. Day 10 post-EVAR CT **c**, **d** shows increased peri-aortic soft tissue with new air foci (block white arrows, **c**, **d**) suggestive of emphysematous infection. The graft was explanted, and microbiology showed *Salmonella enteritidis* graft infection

*Imaging*: CT angiography is the modality of choice for evaluation of EA. Findings include aortic wall thickening, air within or adjacent to the aortic wall, peri-aortic edema, and fat stranding [2, 53] (Fig. 18). EA may be associated with other complications such as a rapidly enlarging mycotic aneurysm, aorto-enteric fistula, or erosion of the adjacent vertebral body [2].

*Prognosis and management:* EA is a severe, life-threatening condition with an overall mortality of 80% without surgical intervention [51, 53]. Management requires intravenous antibiotics and urgent surgical intervention, either resection of the affected native aorta or removal of the infected stent-graft [2].

### Imaging pitfalls of emphysematous aortitis

#### Aorto-enteric fistula

**Fig. 19:**
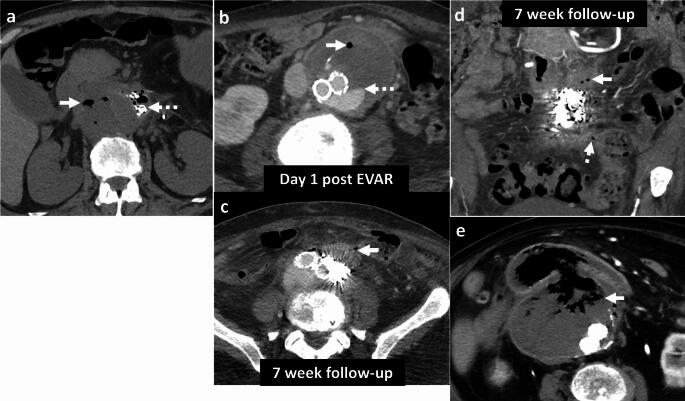
Pitfalls for emphysematous aortitis. **a** Immediate post-surgical changes. A 73-year-old patient status post-infected stent graft explant with placement of antibiotic beads. Immediate post-operative non-contrast CT **a** shows expected peri-aortic fluid and air foci (solid white arrow, **a**) and antibiotic beads (dotted white arrow, **a**). These findings were resolved on follow-up CT. **b**–**d** Immediate post-EVAR changes followed by fistula. An 82-year-old patient status post EVAR with aorto-iliac stent graft. Day-1 post-EVAR CT **b** shows an expected small speck of air (solid white arrow, **b**) in the excluded aneurysm sac. There was a type II endoleak (dotted white arrow, **b**) treated with coil embolization. At 7-week follow-up CT **c**, **d**, there was decreased size of the excluded aneurysm sac but increased peri-aortic air foci (solid white arrow, **c**) and a fistulous connection to colon (dotted white arrow, **d**). The aorto-colonic fistula was confirmed on surgery. The patient underwent stent explant, aortic repair, colectomy and right colostomy. **e** Aorto-duodenal fistula. An 86-year-old post-EVAR and aorto-iliac stent graft, with severe abdominal pain and hematemesis. CT shows direct communication between the excluded aneurysm sac and transverse duodenum (solid white arrow, **e**)

Air can be seen due to communication between the aorta and bowel, most commonly the esophagus, duodenum and small bowel, and colon [54]. Aorto-enteric fistulas can be differentiated from EA by the presence of direct contrast extravasation into the bowel lumen on CTA (Fig. 19). Clinical presentation also differs as EA presents with signs of sepsis, while aorto-enteric fistulas present with gastrointestinal bleeding [54].

#### Post-surgical and post-EVAR changes

These can also demonstrate air and peri-aortic inflammatory changes in the immediate post-operative period (Fig. 19). Correlation with clinical history, serial WBC counts, and resolution of findings on follow-up CT will help differentiate these changes from a developing EA [55].

## Necrotizing retroperitoneal fasciitis and emphysematous iliopsoas abscess

**Fig. 20:**
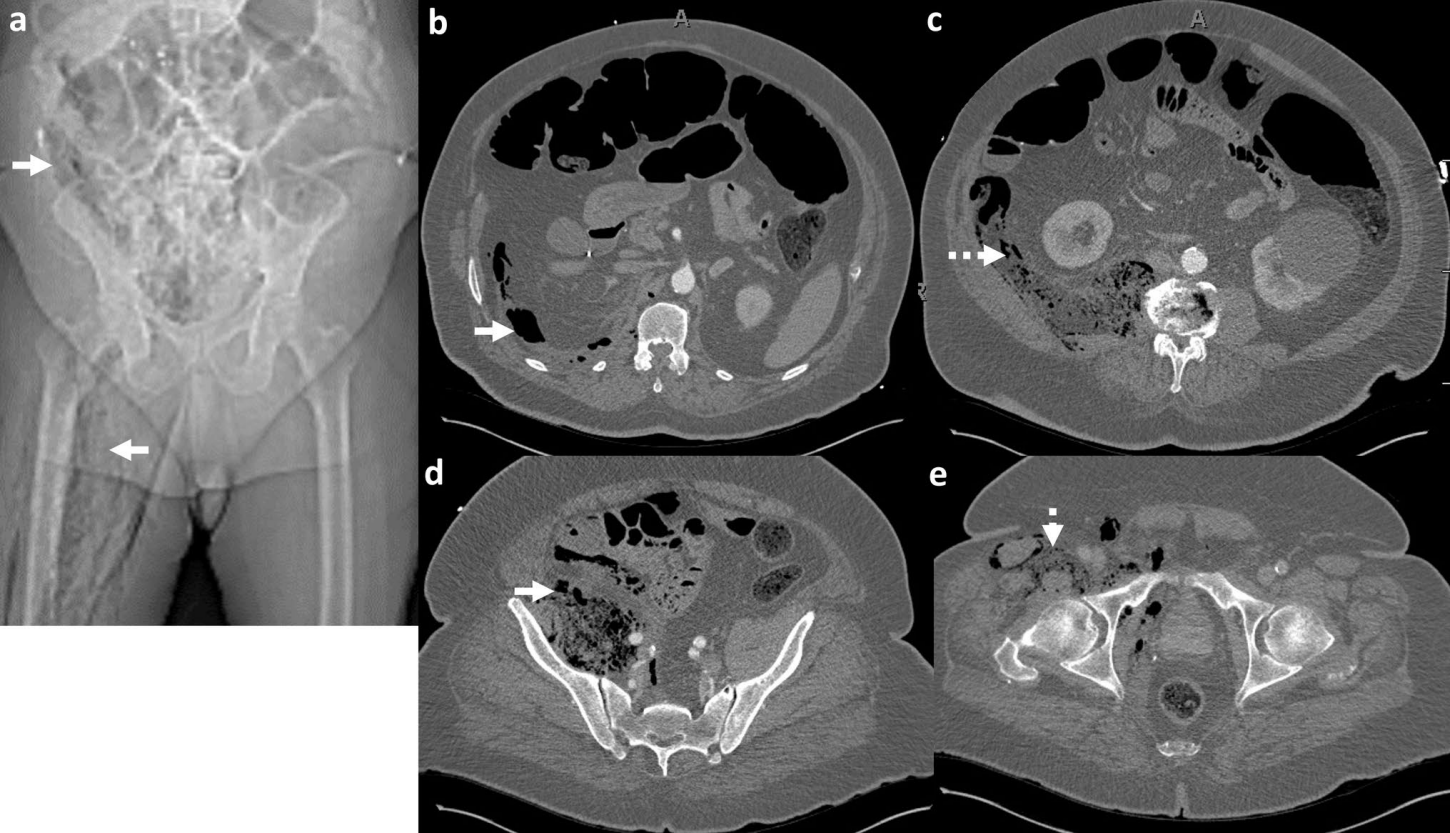
Necrotizing retroperitoneal fasciitis. A 50-year-old diabetic with fever, abdominal, and right leg pain. CT topogram **a** image shows gas outlining muscles of the right thigh and in the right pelvis (white arrows, **a**). CT images **b**–**e** show gas in the right posterior pararenal space (solid white arrow, **b**), right extra-peritoneal pelvis (dotted white arrow,** c**), right iliopsoas muscle (solid white arrow, **d**), and right thigh (dotted white arrow,** e**). The gas transgresses retroperitoneal planes consistent with necrotizing retroperitoneal fasciitis

Necrotizing retroperitoneal fasciitis (NRF) is a rare, gas-forming infection of the retroperitoneal and extra-peritoneal tissues with a potential for rapid progression [56] (Fig. 20). Predisposing factors include diabetes, immunocompromise, obesity, and advanced age [56]. A majority of NRF are associated with an identifiable source of infection from gastrointestinal (GI), genitourinary (GU), traumatic, and iatrogenic causes. Reported sources of infections include diverticulitis, appendicitis, peri-anal abscess, perforated bowel, colon malignancy, pyelonephritis, pancreatitis, spread from complicated pelvic and lower extremity infections, polytrauma, and post-surgical conditions [56–58]. The most common organisms include *β-hemolytic group A Streptococcus*, *Staphylococcus aureus*, polymicrobial infection, and *Mucormycosis* [56]. Clinical presentation is often non-specific, leading to a delayed diagnosis. Symptoms include abdominal pain, flank pain, erythema and tenderness, fever, chills, sepsis, and sometimes, thigh pain secondary to involvement of the psoas muscle [56, 59].

**Fig. 21:**
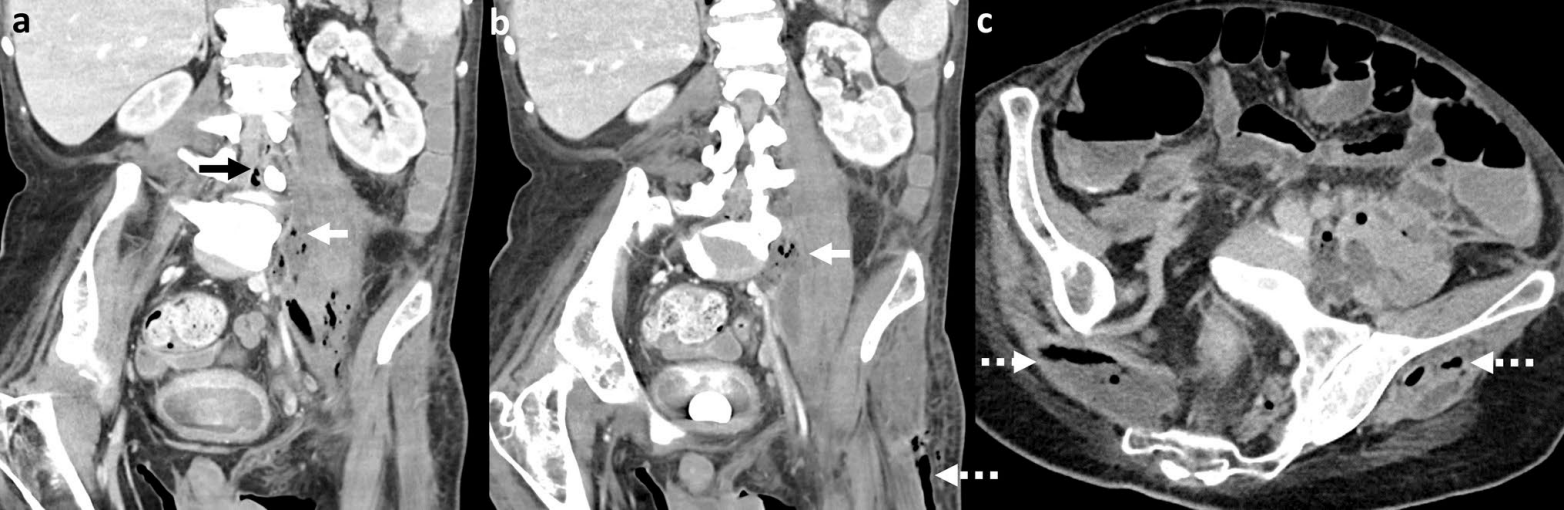
Emphysematous Iliopsoas Abscess. A 44-year-old paraplegic with pressure ulcers, urinary tract infection, and fever. CT images show left iliopsoas abscess with air foci within (solid white arrows **a**, **b**). The abscess extends to the lumbar spinal canal (black arrow, **a**). Air also tracks down to the left thigh (dotted white arrow, **b**), There are additional bilateral gluteal intramuscular abscesses (dotted white arrows, **c**). Percutaneous drain was placed into the iliopsoas abscess, and fluid cultures showed an anerobic polymicrobial infection

Emphysematous iliopsoas abscess (EIA) is a specific form of emphysematous retroperitoneal infection localized to the iliopsoas musculature (Fig. 21). This is usually secondary to colon inflammation or colon cancer. *Clostridium perfringens* and *Clostridium septicum* are the most common causative organisms [2]. EIA can also be multi-compartmental with extension to the pelvis, lower extremity, and spine.

*Imaging*: CT features of retroperitoneal fasciitis include asymmetric fascial thickening, soft tissue and muscle edema, fat stranding, and rim-enhancing fluid collections. Foci of gas tracking across fascial planes is the characteristic feature of necrotizing infection (Fig. 20). CT provides additional information regarding the etiology of necrotizing infection from GI or GU sources [2, 56]. CT can also demonstrate complications such as vascular thrombosis, hemorrhage, organ necrosis, and spinal extension [56] (Fig. 21).

*Prognosis and management:* NRF is a life-threatening condition with high mortality, particularly in patients with underlying risk factors. Expedited surgical management comprising debridement, aggressive parenteral antibiotics/antifungals, and supportive care is crucial. Aspiration of fluid collection is also helpful to guide appropriate antibiotic therapy, and if the diagnosis is uncertain [56, 59].

### Imaging pitfall of necrotizing retroperitoneal fasciitis and emphysematous iliopsoas abscess

#### Pneumoretroperitoneum

**Fig. 22:**
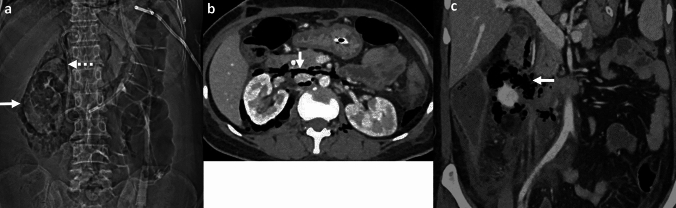
Pneumoretroperitoneum as an imaging pitfall. A 47-year-old with post-ERCP abdominal pain. CT topogram image (**a**) shows veiled kidney sign (solid white arrow, **a**) and ERCP stent (dotted white arrow, **a**). CT images show air in the right retroperitoneum crossing midline (solid white arrows, **b**, **c**). The pneumoretroperitoneum was secondary to duodenal perforation

This refers to gas in the retroperitoneal space with many underlying etiologies. Causes include penetrating trauma, duodenal perforation, perforation of retroperitoneal portions of the colon, extension from pneumomediastinum, and post-procedural changes [60]. Radiographs may show air around kidneys (“veiled kidney” sign), although CT is needed to establish the diagnosis (Fig. 22). It may, however, be difficult to establish whether retroperitoneal air is secondary to NRF or other underlying etiology [60]. A careful assessment of imaging findings and history can help point to the likely etiology. The index of suspicion for necrotizing infection should remain high for acutely ill patients with signs of sepsis, and presence of pneumoretroperitoneum with transgression of fascial planes, and fluid collections on CT [56].

## Emphysematous prostate abscess

Emphysematous prostate abscess is a severe form of prostatitis with a gas-containing collection [61]. Risk factors include diabetes, immunosuppression, bladder outlet and lower urinary tract obstruction, transurethral procedures, and urethral catheterization. Common causative organisms include *Escherichia coli*, *Klebsiella pneumoniae*, *Bacteroides fragilis*, *Citrobacter* species, and *Candida* species [62, 63]. Clinically, patients present with dysuria, frequency, fever, perineal pain, and acute urinary retention. The prostate can be enlarged, tender, and fluctuant on digital rectal examination [61].

**Fig. 23:**
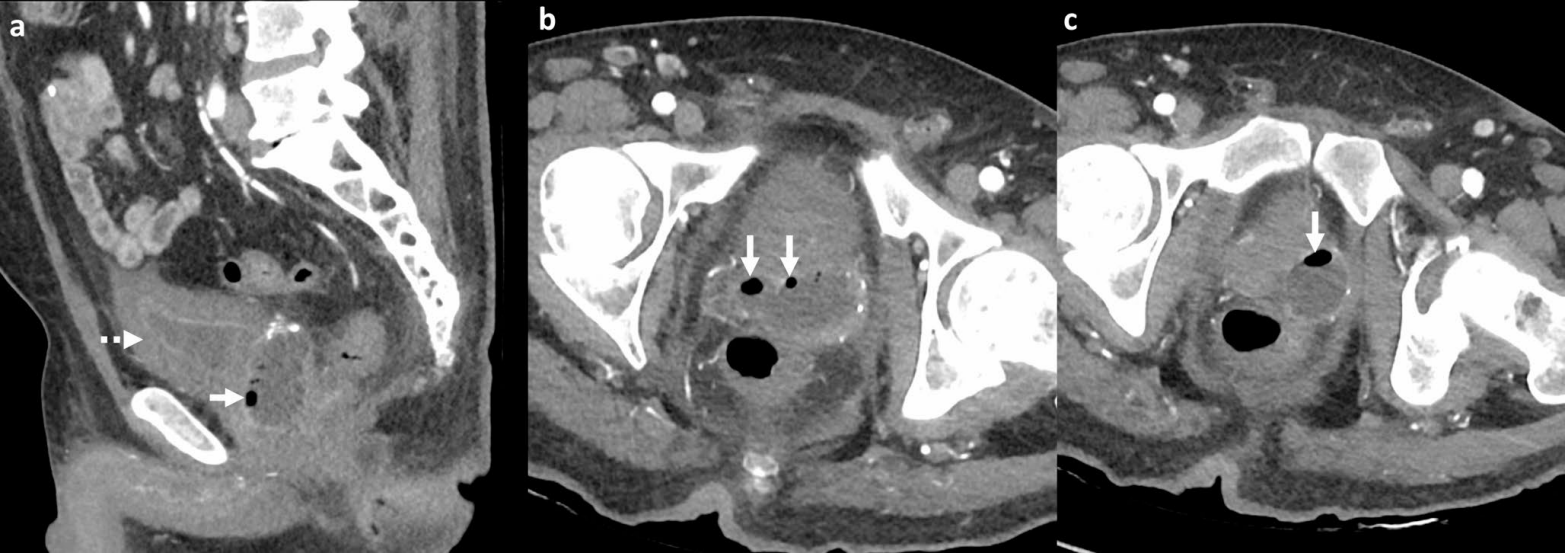
Emphysematous prostate abscess. A 55-year-old with diabetes and end-stage renal disease with fever, dysuria, and bilateral groin pain. CT images show rim-enhancing abscess replacing prostate and bilateral seminal vesicles with air locules (white arrows, **a**–**c**) and changes of cystitis (dotted white arrow, **a**). Blood culture and culture from the abscess showed *Escherichia coli*. The patient was managed with trans-gluteal percutaneous pigtail drain and intravenous antibiotics

*Imaging*: Imaging is crucial for diagnosis due to its non-specific clinical presentation. Radiographs have limited sensitivity and specificity due to the presence of rectal gas [2]. CT can confirm the diagnosis by identifying gas locules in the prostate gland and assess the extent of extra-prostatic inflammation [2, 62] (Fig. 23). Trans-rectal US (TRUS) may show air in the prostate as echogenic foci with posterior shadowing, but these could be mistaken for prostatic calcifications [2].

*Prognosis and management:* The current mortality of emphysematous prostatitis is 3–18% and has decreased compared to the pre-antibiotic era [2, 62]. Management includes drainage, which allows both confirmation of causative organisms to guide therapy and source control [2, 63]. Percutaneous drainage techniques include CT-guided trans-gluteal and US-guided trans-rectal or trans-perineal approaches [62, 64]. Surgical transurethral resection of the prostate can provide complete drainage, but is associated with a higher risk of septicemia [64].

### Imaging pitfalls of emphysematous prostate abscess

#### SpaceOAR infection

**Fig. 24:**
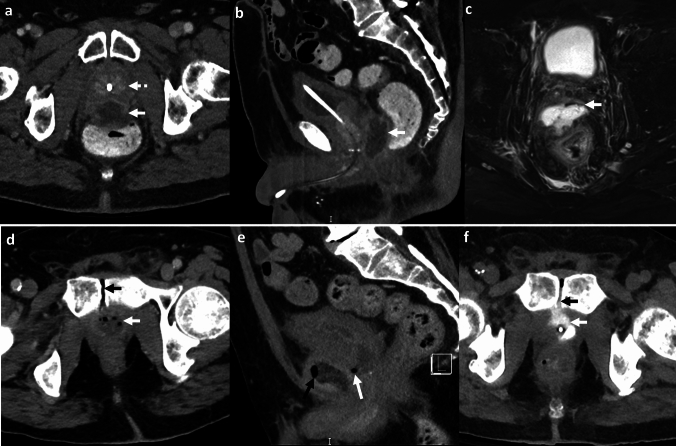
Pitfalls for emphysematous prostate abscess. **a**–**c** Infected SpaceOAR: A 65-year-old with prostate cancer and recent SpaceOAR hydrogel insertion presented with fever and difficulty urinating, CT images **a**, **b** show a rim-enhancing lesion in the recto-prostatic region (solid white arrow, **a**, **b**) separate from the prostate (dotted white arrow, **a**). MRI shows a small air-fluid level anteriorly (solid white arrow, **c**) this was a proven infected SpaceOAR. Erosion into the rectum can also lead to air within the SpaceOAR. **d**–**f** Urosymphyseal fistula: A 71-year-old with prostate cancer and prior radiation therapy with sepsis. CT image **d** shows prostate abscess with air (solid white arrow, **d**, **e**) and air in the pubic symphysis (black arrow, **d**, **e**). Subsequent excretory phase CT image **f** shows excreted urinary contrast filling the prostate cavity (solid white arrow, **f**) and communicating with the pubic symphysis (black arrow,** f**) consistent with urosymphyseal fistula

SpaceOAR hydrogel (SH) is a non-toxic, absorbable substrate that is injected between the rectum and prostate in patients with prostate cancer and helps reduce radiation toxicity to the rectum. SH infection is reported in 8–15% of cases [65, 66]. A history of SH placement and presence of rim-enhancing collection with or without gas locules centered in the retro-prostatic space is helpful in diagnosis (Fig. 24). On MRI, uncomplicated SH is T1W hypointense/T2W hyperintense and does not enhance after contrast [67]. On the contrary, an infected SH shows peripheral enhancement and may be associated with surrounding inflammatory changes. Management requires antibiotics, aspiration, and occasionally surgical removal of the SH [65].

#### Urosymphyseal fistula

This is a rare condition, and typically develops secondary to treatment of pelvic malignancy, including surgery, radiation, or both. Fistula develops between the lower urogenital tract and pubic symphysis, leading to osteitis and osteomyelitis [68, 69]. Patients present with pain on ambulation and chronic urinary tract infections. Imaging is important to establish the diagnosis and assess the extent of the infection. CT demonstrates chronic osteomyelitis with bone erosions, periosteal thickening, sclerotic changes, and pubic diastasis [68]. CT cystogram is occasionally used to demonstrate sinus or fistula tract extending from the bladder or prostate to the pubic symphysis [68] (Fig. 24). MRI is more sensitive to demonstrate altered bone marrow signal, fluid collections, fistulous tracts, and surrounding inflammatory changes on T2W fat-saturated or STIR images [70]. Surgical management includes debridement, cystectomy, or urinary diversion [70].

## Gas gangrene of the uterus

Gas gangrene of uterus (GGU) rarely occurs secondary to septic abortion, septic vaginal delivery, complicated cesarean delivery, puerperal sepsis or trauma. More recently, this has been reported in association with superinfected uterine or cervical malignancy or malignancy with gastrointestinal fistula [2, 71, 72]. Risk factors include complicated labor, maternal diabetes, intrauterine fetal demise, and chorioamnionitis. The most common causative organism is *Clostridium perfringens* [73]. Mixed polymicrobial infection, *Bacteroides* species, *Escherichia coli*, *Streptococcus* species, and *Klebsiella pneumoniae* have also been reported. Clinically, the patients present with fever, pain, purulent vaginal discharge, dysuria or septic shock [72, 73].

**Fig. 25:**
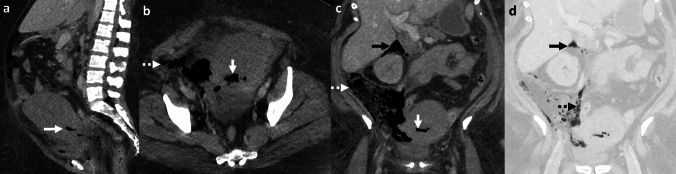
Gas gangrene of uterus. A 35-year-old with intrauterine fetal demise delivered vaginally with chorioamnionitis and puerperal sepsis. CT images show gas in the lower uterine segment (solid white arrows, **a**–**c**), suggesting gas gangrene of the uterus. Gas further extends into the right extraperitoneal pelvis (dotted white arrows, **b**, **c**, and dotted black arrow, **d**) and into the right retroperitoneum (solid black arrows, **c**, **d**), suggesting secondary retroperitoneal fasciitis. The patient underwent hysterectomy and abdominal washout

*Imaging*: Radiographs may show mottled gas in the pelvis outlining the uterus. On US, GGU appears as uterine enlargement along with echogenic foci with dirty shadowing. CT is the most accurate modality to establish the diagnosis by confirming the presence of gas within the uterine cavity extending to the myometrium (Fig. 25). In severe cases, gas and fluid collections may also further extend to the peritoneal cavity or extraperitoneal spaces, leading to peritonitis and necrotizing fasciitis, respectively [2]. CT can also demonstrate underlying gynecologic malignancy and bowel fistula.

*Prognosis and management:* Early initiation of intravenous antibiotics is crucial for the treatment of GGU. Dilation and curettage may be performed if there is limited endometrial involvement. Hysterectomy and surgical debridement are indicated when there is involvement of myometrium (myonecrosis), extra-uterine extension, or underlying malignancy [2]. Reported mortality ranges between 20 and 85%, but has decreased with early surgical management [71, 72].

### Imaging pitfalls of gas gangrene of the uterus

#### Post-embolization changes

**Fig. 26:**
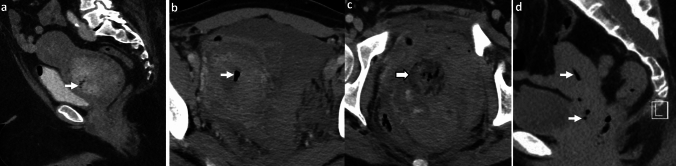
Pitfalls for gas gangrene of the uterus. **a** Post-embolization changes. CT image shows expected air foci within cervical fibroid post-uterine artery embolization (solid white arrow, **a**). **b**, **c** Post-partum and surgical changes. Patient with full-term vaginal delivery complicated by vaginal hematoma, post incision-drainage and hemostatic packing. CT images show expected post-partum air in the uterine cavity (solid white arrow, **b**) and para-uterine lucencies secondary to hemostatic pack (block white arrow, **c**). **d** Post-procedure changes. Air in the endometrial cavity and endocervical canal (solid white arrows,** d**) after removal of the intrauterine device

Air can be seen within the uterine myometrium after uterine artery embolization for uterine or cervical fibroids (Fig. 26). Patients may occasionally present with PES clinically. Both symptoms and imaging findings resolve on follow-up [74].

#### Normal post-partum and post-procedural changes

Presence of gas in the endometrial cavity has been typically associated with endometritis. However, around 20% of uncomplicated deliveries may have endometrial air on imaging [75]. Similarly, uterine procedures such as dilation and curettage and cesarean section can have air in or around the uterus (Fig. 26). Careful correlation with medical records and clinical symptoms is crucial for an accurate diagnosis.

## Vaginitis emphysematosa

Vaginitis emphysematosa (VE) is a benign, self-limiting condition characterized by air-filled submucosal cysts in the vagina and or exocervix [76]. The pathology of VE is not completely elucidated, but has been associated with *Trichomonas vaginalis*, *Hemophilus vaginalis,* and *Gardnerella vaginalis* infection in immunosuppressed or pregnant patients [76, 77]. Clinical features include pruritus, vaginal discharge, and occasionally vaginal fullness and crepitus during intercourse. Cysts may be seen and palpated on physical exam with speculum and most often found in the upper vagina and/or cervix [76].

**Fig. 27:**
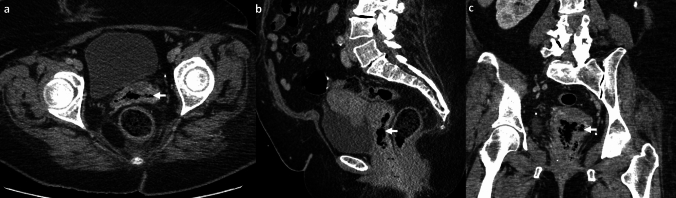
Vaginitis emphysematosa (VE). Incidental findings of air-filled cysts in vaginal wall (white arrows** a**–**c**). Note the submucosal location in vaginal wall best seen on the coronal images (Image courtesy Reddy R, MD)

*Imaging*: Radiographs may show gas locules or bubbles clustered along the vaginal wall. Transvaginal ultrasound shows echogenic air foci uniformly spread throughout the vaginal wall. CT shows gas in the vaginal wall in a pattern similar to that of pneumatosis intestinalis (Fig. 27). Gas is commonly found in the upper two-thirds of the vagina but can extend to the lower vaginal wall and occasionally introitus and labial folds [77].

*Prognosis and management:* VE is not typically associated with necrosis or life-threatening infection, and therefore, expectant management can be pursued until resolution of symptoms. Alternatively, wet mount for microbial confirmation and treatment with antibiotics such as metronidazole can be considered [76].

### Imaging pitfalls of vaginitis emphysematosa

#### Intravaginal gas

**Fig. 28:**
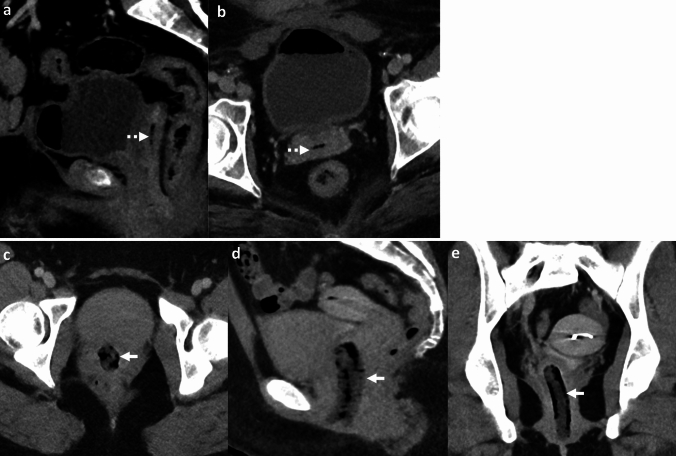
Pitfalls for air in vagina. **a**, **b** Intravaginal gas: Patient with hysterectomy and cystitis with recent Foley’s catheterization. CT shows trace air within remnant vaginal canal (dotted white arrow, **a**, **b**). **c**–**e** Vaginal tampon: A 21-year-old with abdominal pain. CT images show incidental vaginal tampon (solid white arrows, **c**–**e**). Mottled lucencies are due to air trapped in the cotton/cellulose material of the tampon

Gas within the vaginal canal is a commonly seen as a non-specific finding on CT. This usually has no clinical significance in the absence of an underlying fistula, malignancy, or pelvic infection (Fig. 28).

#### Vaginal tampon

This is another common cause of mottled air in the vaginal region. However, the tampon is recognized by its distinct geometric shape (Fig. 28).

## Necrotizing fasciitis of the abdominal wall and Fournier's gangrene

Necrotizing fasciitis of the abdominal wall (NFAW) or gas gangrene of the abdominal wall is a fulminant gas-forming infection of the abdominal and pelvic wall [2]. Fournier's gangrene (FG) specifically refers to necrotizing gas-forming infection of the perineal, perianal, and scrotal soft tissues [2]. NFAW and FG are often polymicrobial or due to *Clostridium* species. These conditions have a strong male predisposition and are more common after the 5th decade of life [78]. Most patients have underlying comorbidities such as obesity, diabetes, immunosuppression, and peripheral vascular disease [78]. Other predisposing factors include trauma, surgery, malignancy, and spread from adjacent infections. FG is associated with an untreated peri-anal fistula/abscess and, rarely, rectal malignancy [79]. NFAW is associated with underlying GI conditions such as diverticulitis, perforated appendicitis, pancreatitis, complicated Crohn’s disease, and perforated colorectal cancer [80]. Clinically, patients present with “dishwasher”-like skin discoloration, erythema, pain, swelling, discharge, fever, and sepsis [81]. Upon palpation, crepitus is felt in the affected region [82].

**Fig. 29:**
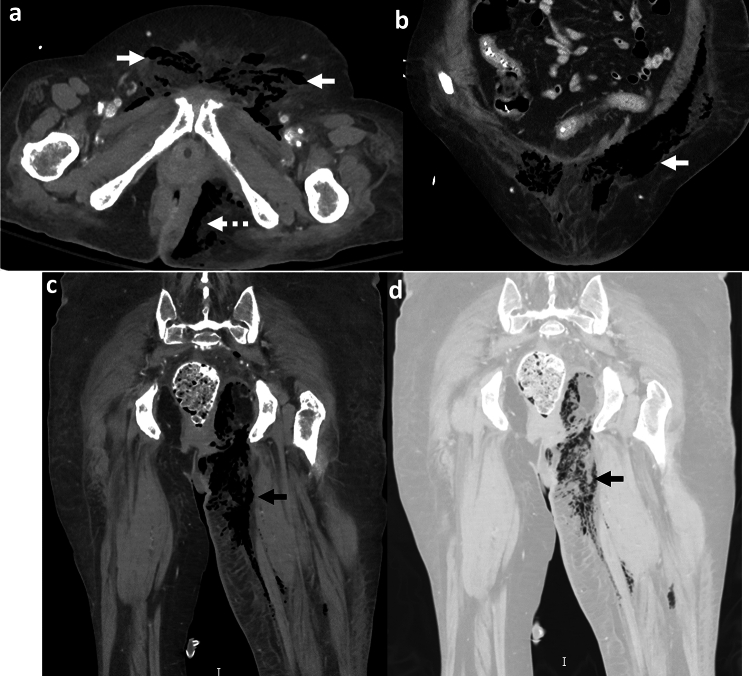
Necrotizing fasciitis of abdominal wall. A 77-year-old with diabetes, dysuria, fever, and pain. Clinical exam showed edema and erythema in the perineal region and left thigh with skin necrosis. CT images show subcutaneous air with fat stranding and fascial thickening in the anterior abdominal wall (solid white arrows, **a**,**b**), left gluteal region (dotted white arrow, **a**), and left upper thigh (black arrow,** c**,**d**). Surgical debridement was performed. Cultures showed admixed *Clostridium*, *Bacteroides, Streptococcus*, *Staphylococcus* species, and anaerobes. The patient expired because of fulminant sepsis

**Fig. 30:**
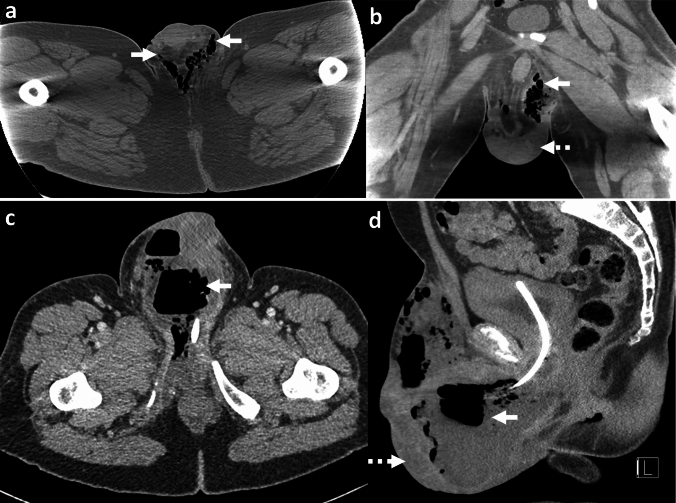
Fournier’s gangrene. **a**, **b** A 35-year-old diabetic with scrotal pain and erythema. CT images show air in the scrotum (solid white arrows, **a**, **b**) with scrotal skin thickening (dotted white arrow, **b**). **c**, **d** A 55-year-old diabetic with urosepsis, scrotal swelling, pain, and erythema. CT images show air in the scrotum with multiple air-fluid levels (solid white arrow, **c**) with extensive scrotal wall edema (dotted white arrow, **d**)

*Imaging*: Radiographs may demonstrate soft tissue air locules; however, they have poor sensitivity for evaluation of subcutaneous emphysema [82]. Similarly, US may be used as a screening modality and can demonstrate fasciitis (thickening of fascia > 4 mm), echogenic collections along deep fascia, myositis, and echogenic foci due to gas [81, 83]. CT readily identifies subcutaneous emphysema as locules of air dissecting along the subcutaneous fascial planes (Fig. 29). Decreased muscle enhancement due to myonecrosis, fascial thickening, fat stranding, fluid collections, and abscess may also be evident on CT[83] (Fig. 30). MRI is the most sensitive imaging modality to demonstrate multicompartmental involvement, soft tissue edema, fluid collections, and tissue necrosis, but is difficult to perform in an acute setting [81, 83].

*Prognosis and management:* Mortality of NFAA and FG remains variable, ranging between 30 and 60% despite early diagnosis and surgical management [78, 81]. Early initiation of parenteral antibiotics, fasciotomy, and surgical debridement of necrotic tissues are the mainstay of management. Treatment with hyperbaric oxygen is associated with improved survival [84].

### Imaging pitfalls of necrotizing fasciitis of the abdominal wall and Fournier's gangrene

#### Abdominal/scrotal air due to trauma or surgery

**Fig. 31:**
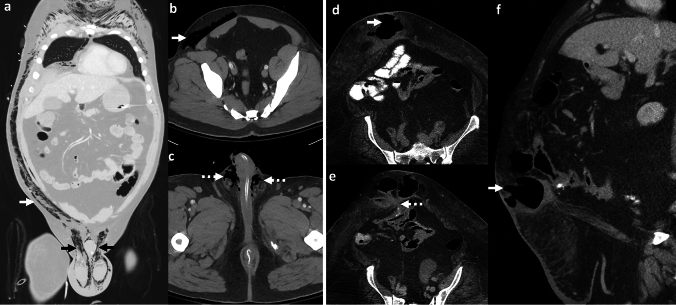
Pitfalls for Necrotizing fasciitis of abdominal wall and Fournier's gangrene. **a**–**c** Post-trauma or surgery: A 24-year-old with trauma and right flail chest. CT images show extensive subcutaneous emphysema (solid white arrow, **a**,**b**) with air tracking down to the scrotum outlining bilateral inguinal scrotal regions (black arrows, **a**; dotted white arrows, **c**). Unlike necrotizing fasciitis, air respects fascial planes, and there are no associated inflammatory changes. **d**–**f** Enterocutaneous fistula: A 57-year-old with swelling in the anterior abdominal wall. CT shows air fluid levels in the anterior abdominal wall with surrounding fat stranding (solid white arrows, **d**, **f**) and underlying enterocutaneous fistula (dotted white arrow, **e**)

Air can normally be introduced into subcutaneous soft tissues following trauma or secondary to surgical intervention (Fig. 31). In contrast to necrotizing infections, iatrogenic air usually respects fascial planes with no or minimal inflammatory changes. There can be imaging overlap between traumatic subcutaneous emphysema and necrotizing fasciitis, therefore, clinical correlation and close follow-up are required to exclude superimposed necrotizing infection in polytrauma patients [81].

#### Enterocutaneous fistulas

These can also introduce air into the abdominal wall soft tissues but are often clinically suspected. Other imaging studies, such as CT fistulogram, CT with IV and oral contrast, and MR enterography can confirm the diagnosis and assess the anatomic origin of the gas in the abdominal wall [85] (Fig. 31).

## Emphysematous epididymo-orchitis

Emphysematous epididymo-orchitis (EEO) is a rare condition characterized by gas-forming infection of the testicle and/or epididymis [86]. This is unlike FG, where gas is seen in the scrotum and perineum. EEO is usually seen in men above the age of 50 with comorbid conditions such as diabetes, immunocompromised status, and urinary tract infection. The most common mechanism is ascending bacterial infection from the urethra or the urinary bladder [86]. Causative organisms include *Escherichia coli*, *Klebsiella pneumoniae*, and, less commonly, *Pseudomonas*, *Proteus*, and *Bacteroides* species [86, 87]. Clinically, EEO has a nonspecific presentation with scrotal pain, tenderness, swelling, erythema, and fever.

**Fig. 32:**
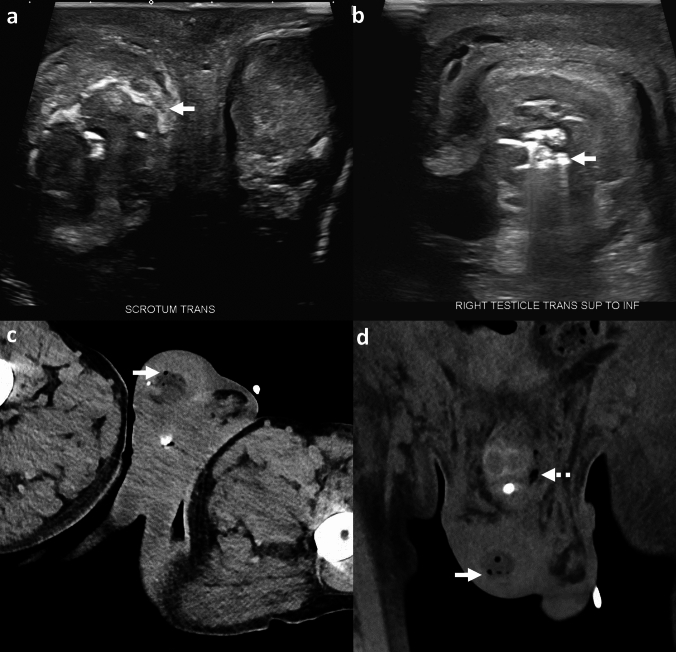
Emphysematous epididymo-orchitis. A 54-year-old with a foreign body insertion into the urethra, presenting with scrotal pain and difficulty voiding. Transverse US images show air within the enlarged right testis, seen as echogenic foci with dirty posterior acoustic shadowing (solid white arrow, **a**, **b**). CT images show air within the right testis (solid white arrows, **c**,** d**) with scrotal edema. Also note air in the penile shaft secondary to penetrating urethral injury (dotted white arrow, **d**). The patient underwent surgical debridement. Cultures showed *Escherichia coli*, *Proteus mirabilis*, *Streptococcus* species, and anaerobes

*Imaging*: Imaging plays a crucial role in establishing the diagnosis, given the non-specific clinical presentation. US of the scrotum is usually the first modality to assess scrotal infections. UUS may show enlargement and altered heterogenous echotexture of the testis and/or epididymis [87]. Intratesticular air, seen as scattered echogenic foci with dirty posterior acoustic shadowing, is the key diagnostic feature (Fig. 32). Pelvic radiographs may show air locules in the topography of the scrotum but have poor specificity. CT is the most sensitive modality to confirm the presence of air within the testis/epididymis and exclude other differential diagnoses [87].

*Prognosis and management:* EEO is a potentially lethal infection, but overall has a better outcome than FG [86]. Treatment includes intravenous antibiotics and prompt surgical debridement with possible orchiectomy. If left untreated, complications include dissemination of infection into adjacent soft tissues leading to fulminant necrotizing fasciitis of the penis and/or FG [86, 87].

### Imaging pitfalls of emphysematous epididymo-orchitis

#### Echogenic intratesticular foci on ultrasound

**Fig. 33:**
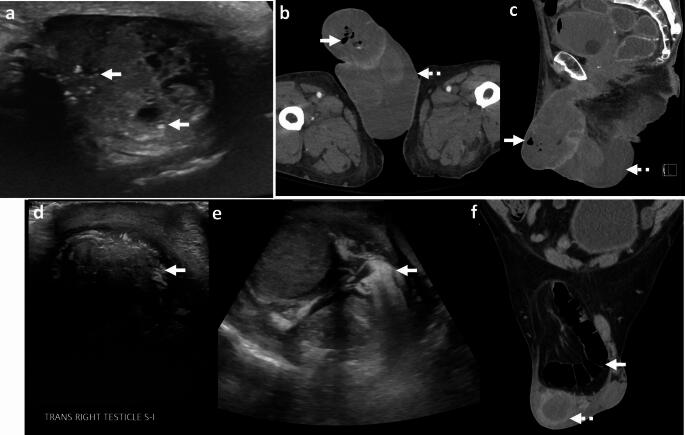
Pitfalls for emphysematous epididymo-orchitis. **a** Intratesticular echogenic foci: A 28-year-old with right testicular lump. US shows heterogenous mass with necrosis and punctate echogenic foci due to calcifications (solid white arrows, **a**). Pathology showed yolk sac tumor. **b**, **c** Necrotic tumor. A 65-year-old with penile cancer with air foci (solid white arrow, **b**, **c**) due to post-radiation tissue necrosis and scrotal wall edema (dotted white arrows, **b**,** c**). **d**–**f** Inguinoscrotal hernia. A 54-year-old with right scrotal erythema and swelling. Transverse US images show air foci with dirty posterior acoustic shadowing in the right scrotum (solid white arrows, **d**, **e**) and a fluid collection (not shown). CT image shows right inguinoscrotal hernia containing colon (solid white arrow, **f**) and incidental abscess in right hemiscrotum (dotted white arrow, **f**). The air foci seen on US were due to herniated bowel and were not related to the incidental scrotal abscess

Several entities may demonstrate echogenic foci in the testis on US, including testicular lipomatosis, testicular microlithiasis, testicular penetrating trauma with shrapnel, and testicular tumors with calcifications [88] (Fig. 33). Lack of acute illness and absence of signs of infection and sepsis are key to establishing the diagnosis.

#### Bowel containing inguinoscrotal hernia

Bowel containing inguinoscrotal hernia may also present with foci of air within the scrotum. However, the bowel segment is adjacent to and possibly displacing the testis and is readily identified on CT [89] (Fig. 33).

#### Malignancy

Air can develop within genital tumors due to tissue breakdown or post-radiation necrosis (Fig. 33). Again, lack of acute presentation helps differentiate malignancy with gas from a more fulminant necrotizing infection.

## Conclusion

Emphysematous conditions share common predisposing factors. Apart from vaginitis emphysematosa, the entities discussed in the article represent potentially life-threatening conditions necessitating urgent imaging and multidisciplinary care. As shown in this review, imaging pitfalls containing gas are commonly due to iatrogenic causes, trauma, or fistulae. A careful review of CT images with adequate windowing and appropriate correlation with history and laboratory results are essential to avoid misdiagnosis.

## Data Availability

No datasets were generated or analysed during the current study.
